# Health assessment and multipathogen surveillance of free-ranging snakes in the southeastern United States

**DOI:** 10.3389/fvets.2026.1754420

**Published:** 2026-05-26

**Authors:** Corinna M. Mishin, Terence M. Farrell, Jenna N. Palmisano, Robert J. Ossiboff, Makaylah McCray, Ellen Haynes, John C. Maerz, Kristina Meichner, Jian Zhang, Kayla B. Garrett, Michael J. Yabsley, Jason Ortega, Christopher A. Cleveland, Nicole M. Nemeth

**Affiliations:** 1 Southeastern Cooperative Wildlife Disease Study, Department of Population Health, College of Veterinary Medicine, University of Georgia, Athens, GA, United States; 2 Department of Biology, Stetson University, DeLand, FL, United States; 3 Department of Biology, University of Central Florida, Orlando, FL, United States; 4 Department of Comparative, Diagnostic, and Population Medicine, College of Veterinary Medicine, University of Florida, Gainesville, FL, United States; 5 Warnell School of Forestry and Natural Resources, University of Georgia, Athens, GA, United States; 6 Department of Pathology, College of Veterinary Medicine, University of Georgia, Athens, GA, United States; 7 Center for the Ecology of Infectious Diseases, University of Georgia, Athens, GA, United States; 8 Department of Biological Sciences, University of Arkansas—Fort Smith, Fort Smith, AR, United States

**Keywords:** *Cryptosporidium*, *Hepatozoon*, *Mycoplasma*, *Ophidiomyces ophidiicola*, *Raillietiella orientalis*, *Salmonella*, serpentovirus, snake health

## Abstract

**Introduction:**

Health assessments and pathogen surveys in wildlife are critical for gauging population-level risks. Recent concerns associated with ophidiomycosis, caused by the fungus *Ophidiomyces ophidiicola* (*Oo*), have brought needed attention to snake conservation.

**Methods:**

We evaluated the health of native snake species in the southeastern United States through physical examinations and multipathogen surveillance targeting *Cryptosporidium* spp., *Hepatozoon* spp., *Mycoplasma* spp., *Oo*, *Raillietiella orientalis* (*Ro*)*, Salmonella* spp., and serpentoviruses. We hypothesized that apparent ophidiomycosis (skin lesions present and qPCR detection of *Oo*) would be positively associated with coinfection detection, and that pathogen detection prevalence would vary spatiotemporally and among snake species. We assessed pathogen and disease prevalence through monthly sampling from May 2022 to May 2024 (via full-body skin swabs, choanal swabs, cloacal swabs, blood, and fecal collection, and physical examinations) at wetland sites in Volusia County, Florida, and Jasper County, South Carolina. We opportunistically sampled snakes from a site in Athens-Clarke County, Georgia, and included diagnostic cases submitted to the Southeastern Cooperative Wildlife Disease Study from 2021 to 2024.

**Results:**

In total, we sampled 509 individuals with 49 recaptures, representing 29 species. We included 61 carcasses, comprising 56 individuals (33 collected opportunistically) and five recaptures. We detected *Salmonella enterica* in 62.6% (306/489), *Hepatozoon* spp. in 53.4% (205/384), *Mycoplasma* spp. in 17.5% (78/445), *Oo* in 16.1% (82/508), and *Cryptosporidium* spp. in 2.0% (10/489). Detection of *Ro* was limited to snakes in Florida, with a detection prevalence of 12.7% (37/292). No serpentoviruses were detected (*n* = 447). Overall coinfection prevalence was 44.0% (219/498) and was strongly predictive of apparent ophidiomycosis (*p* < 0.0001). *Mycoplasma* spp. and *Cryptosporidium* spp. were nearly exclusively detected in snakes from Florida. Seasonal trends were seen in the detection of *Mycoplasma* spp. (*p* = 0.0075), *Oo* (*p* = 0.0475), and *S. enterica* (*p* = 0.0295). Detection of *Oo* (*p* = 0.0002) or *Ro* (*p* = 0.0200) was negatively associated with nutritional condition scores. Increased detection risks of *Oo* (*p* = 0.0479) and apparent ophidiomycosis (*p* = 0.0148) were observed primarily in pygmy rattlesnakes (*Sistrurus miliarius*), supported by severe associated pathology, indicating increased conservation risks.

**Discussion:**

This health assessment enhances understanding of infectious agents circulating in free-ranging snakes in the southeastern United States and establishes baseline parameters to inform conservation management.

## 1 Introduction

Emerging infectious diseases of wildlife are a significant threat to global biodiversity, wildlife population health, and, in some cases, human and domestic animal health (1). Globally, approximately 21% of reptile species are threatened with extinction, which is likely to be exacerbated by disease (2, 3). In 2013, the ascomycetous fungal etiologic agent of ophidiomycosis, *Ophidiomyces ophidiicola* (*Oo*), was formally described and is now recognized as a global fungal disease of snakes (4–6). Ophidiomycosis has been associated with population declines in conservation-priority species [e.g., the endangered eastern massasauga rattlesnake (*Sistrurus catenatus*)], and *Oo* has since been detected in more than 60 snake species across multiple continents (7–12). Clinical presentation of ophidiomycosis typically consists of cutaneous lesions (e.g., misshapen scales, crusting, ulcerations, etc.) and, when severe, can be disfiguring and/or may disseminate to internal organs and cause death (13–16).

Ophidiomycosis exhibits a complex ecoepidemiology driven by a multifaceted interplay of factors in the epidemiologic triangle, including environmental factors (e.g., season, geography, habitat type, climate), host factors (e.g., taxonomy, life history, age, immune status, concurrent infections, past trauma, reproductive status, brumation behaviors, host microbiome), and pathogen factors (e.g., strain, optimal growth conditions) (17–28). All snake species are considered susceptible to ophidiomycosis; however, there are important species differences in the risk of severe ophidiomycosis development that require further evaluation (11, 18, 29–36). Ophidiomycosis can further lead to increased rates of ecdysis, increased time spent basking, and altered microbiome composition, which are important defense mechanisms against invading pathogens (25–27, 37–44). These changes may initially facilitate infection clearance but can ultimately lead to immunosuppression through increased energetic costs (25–27, 37–45).

The potential role of coinfections in the development of severe ophidiomycosis and their broader impacts on free-ranging snake health remain poorly understood. Moreover, information on many common global and/or endemic snake pathogens is lacking. These include *Salmonella enterica*, a bacterium that is considered normal reptilian flora but can cause fatal salmonellosis in snakes (46–52). Additionally, *Hepatozoon* spp. are genetically diverse intraerythrocytic apicomplexan protozoans that are common in free-ranging snakes globally and are generally considered subclinical (53–56). Another noteworthy opportunistic pathogen in snakes, which has yet to be investigated in wild populations, is *Mycoplasma* spp., a group of bacteria characterized by their lack of a cell wall and classified within the class Mollicutes (57). A recently introduced threat to snake health in the southeastern United States is the hematophagous pentastome *Raillietiella orientalis* (*Ro*), likely introduced by invasive Burmese pythons (*Python bivittatus*) in south Florida (58–60), and which is now rapidly spreading among native snakes with associated mortalities (61, 62). Serpentoviruses, in the order *Nidovirales*, are an established threat in captive snakes but have also been recently detected in native free-ranging snakes in south Florida [two brown watersnakes (*Nerodia taxispilota*), two Florida green watersnakes (*Nerodia floridana*), and a corn snake (*Pantherophis guttatus*)] (63). *Cryptosporidium* spp. are gregarine apicomplexan protozoan parasites with significant health implications in captive snakes (64). *Cryptosporidium serpentis* has been detected at low prevalence in native, free-ranging snakes, specifically the threatened eastern indigo snake (*Drymarchon couperi*), warranting further investigation in wild populations (65–68). Health assessments of free-ranging snakes in the southeastern United States have thus far been limited to a single species, the eastern indigo snake (65, 67). These assessments provide key insights, including high pathogen detection prevalence (*Ro* and *Cryptosporidium* spp.), suggesting heightened transmission rates that may be facilitated by stress-induced immunosuppression (65, 67).

Herein, we aim to establish a foundation for holistic snake health assessments that can inform conservation management by characterizing infectious agents present in free-ranging populations with potential health implications. Our study objectives were to (1) conduct multipathogen surveillance and physical examinations in free-ranging southeastern US snakes at two wetland sites; (2) compare pathogen prevalence over time, among snake species, and between study sites; and (3) assess associations between ophidiomycosis severity, multipathogen detections, and demographic and spatiotemporal factors. We hypothesized that cases of apparent ophidiomycosis (skin lesions present and qPCR detection of *Oo*) would be positively associated with the detection of coinfections and that the prevalence of detected pathogens would vary spatiotemporally and among snake species.

## 2 Methods

### 2.1 Animal capture and sample collection

We captured snakes using a variety of field methods, including visual encounter, road cruising, cover board, and minnow trap surveys. We targeted all native snake species to maximize sample size and species diversity. We conducted surveys at two National Wildlife Refuges, one in Jasper County, South Carolina, and the other in Volusia County, Florida, monthly from May 2022 to May 2024 (approximately 24 months; US Fish and Wildlife Service Special Use Permit #2022-24 and MI-2-21-207R). Field methods for both sites included visual encounter and minnow trap surveys; however, road cruising and cover board surveys were primarily utilized at the South Carolina site (based on the site’s landscape characteristics). Additionally, opportunistic sampling from 2021 to 2024 included snake carcasses found on roads, carcasses submitted to the Southeastern Cooperative Wildlife Disease Study Research and Diagnostic Service, and live snakes captured in Athens-Clarke County, Georgia, by the same means as described above (Georgia Department of Natural Resources Scientific Collection Permit Order #120250674, 121599430, 122970844, and 124108785). Protected species [e.g., eastern indigo snake (*Drymarchon couperi*)] were only included when sampled and/or submitted by state or federal wildlife agencies. Specific site names and locations are withheld to prevent illegal snake collection.

**Figure 1:**
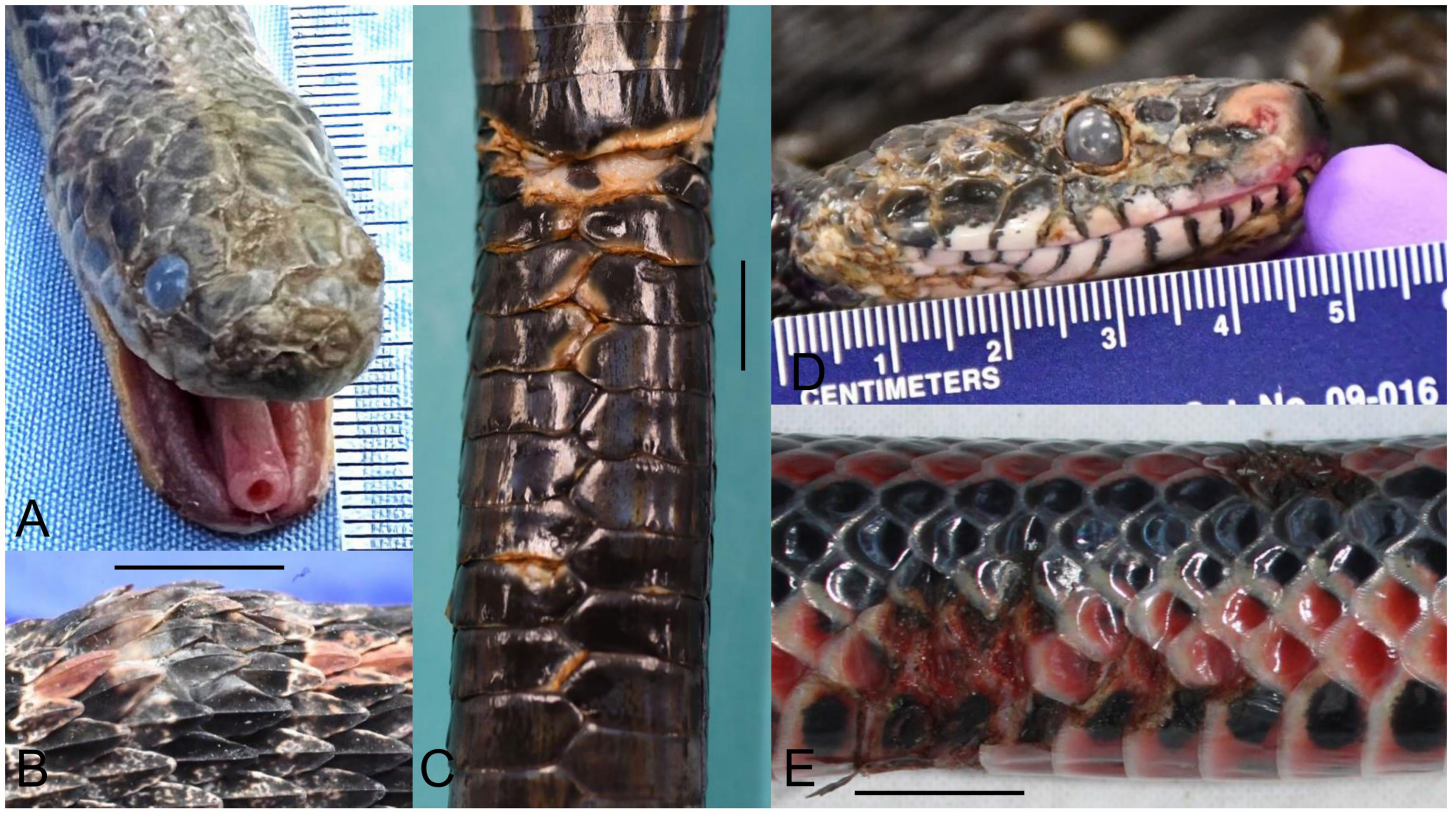
Example skin lesions associated with ophidiomycosis. **(A)** Open-mouth breathing in a rainbow snake (*Farancia erythrogramma*), along with rostral skin crusting and misshapen scales. **(B)** Focal dermal swelling and crusting of overlying dorsal scales in a pygmy rattlesnake (*Sistrurus miliarius*). **(C)** Severe, multifocal scale ulceration and shrinkage with yellow-brown, devitalized margins on the cloaca and ventral tail of an eastern indigo snake (*Drymarchon couperi*). **(D)** Diffuse crusting on the head skin with focal rostral ulceration and retained ocular sclera in an eastern rat snake (*Pantherophis alleghaniensis*). **(E)** Multifocal lateral body skin ulceration, misshapen scales, and overlying dried hemorrhage in a rainbow snake. Scale bar = 1 cm.

At capture, we recorded the date, time, state, county, latitude, and longitude of the location, as well as environmental data (temperature and relative humidity). Temperature and relative humidity at the point of first observation were determined using a handheld Kestrel^®^ device (Kestrel^®^ 3500 NV Weather Meter, Kestrel^®^ Instruments, Boothwyn, PA). We placed non-venomous snakes in clean cloth bags [i.e., snake bags (Midwest Tongs Inc., Greenwood, MO) or pillowcases] and venomous snakes in clean plastic 5-gallon buckets with secured lids and holes for ventilation. Generally, within 6 h (and rarely up to 24 h) of capture, physical examinations were conducted in the field or in the field laboratory to collect demographic and morphological data [species, snout–vent length (SVL), total length, age class, mass, sex], shed status, and health parameters (nutritional condition score, mentation, signs of upper respiratory infection, and gross skin lesions; Figure 1). Ordinal nutritional condition scores were assigned on a 1–5 scale with 0.5 step increments (to account for intermediate classifications) based on physical examination evaluating rib and spinal prominence and epaxial muscle volume, as further outlined in Table 1 (69). For each skin lesion, we recorded the surface area affected (in mm), anatomical location, and lesion type (e.g., misshapen scales, surface scabbing or crusting, and ulceration). These criteria were then used to assign an ordinal lesion severity score ranging from 4 to 12, calculated as the sum of points across four categories, as outlined in Table 2 (Figure 1) (14). Snakes without lesions received a score of 0. All nutritional conditions and lesion severity scores were assigned by the same observer (CMM). We further documented any clinical signs, including those suggestive of upper respiratory disease, such as open-mouth breathing or excessive mucus in the nares and/or oral cavity.

**Table 1:** Ordinal nutritional condition scoring system adapted from Gimmel et al. (69), used to examine free-ranging snakes in the southeastern United States from 2021 to 2024.

Nutritional condition scores	1/5	2/5	3/5	4/5	5/5
Spine	Sharp, no musculature	Easily palpable, minimal musculature	Palpable, musculature evident	Palpable with pressure	Not palpable
Ribs	Sharp and prominent	Easily palpable	Palpable	Palpable with pressure	Not palpable
Neck	Slender	Slender	Straight	Broad	Broad

Scores were recorded on a scale of 1–5 with 0.5-step increments.

**Table 2:** Gross skin lesion severity scoring system adapted from Baker et al. (14), used to examine free-ranging snakes in the southeastern United States from 2021 to 2024.

Lesion category	1 point	2 points	3 points
Type	Displaced or abnormal scale	Scab, crust, and/or nodule	Ulcer
Location	Body or tail	Head (except mouth, nasolabial pits, or eyes)	Mouth, nasolabial pits, eyes, cloaca
Quantity	1	2–4	≥5
Size	<10 mm	10–50 mm	≥50 mm

Scores were calculated as the sum of points across the four categories. Scores ranged from 4 to 12.

Full-body skin swabs were collected using cotton-tipped applicators (Fisherbrand^™^ Plastic Handled Cotton Swabs and Applicators, Thermo Fisher Scientific Inc., Waltham, MA) by thoroughly, bidirectionally swabbing the head, dorsum, and lateral aspects of the body and tail, and unidirectionally (with scales) swabbing the ventrum, taking care to include any grossly evident skin lesions (14, 70). Cloacal swabs (Sterile Rayon Tipped Applicators, Puritan^®^ Medical Products, Guilford, ME) were collected by inserting a swab into the cloaca and slowly rotating it approximately five full turns. The choana was sampled by inserting a swab (swab brand selected based on snake size) into the oral cavity and placing the swab directly into the choana and rotating it approximately five full turns. We did not collect choanal swabs from live venomous snakes due to safety concerns. Feces were opportunistically collected either upon voluntary defecation (e.g., in defense) or by manual extraction through gentle palpation of the rectum.

Blood samples were collected via venipuncture (≤0.5 mL of blood; no more than 0.8% of body weight) from snakes of adequate size (approximately >15 g) from the ventral coccygeal vein, immediately placed into lithium-heparinized microtainer tubes (Becton Dickinson, Franklin Lakes, NJ), and stored on ice packs while in the field and during transport. Blood films were made immediately following collection using fresh, non-heparinized blood, except when inclement field conditions required placing the entire blood volume into the heparin microtainer, in which case blood film creation was delayed and films were made with heparinized blood. Slides were then stained with Hema 3^™^ Stat Pack (Thermo Fisher Scientific, Waltham, MA). Parasites (e.g., oral trematodes, mites, leeches, etc.) observed during physical examination were opportunistically collected and preserved in 70% ethanol. Snakes of adequate size (estimated to be >15 g) were marked using a Passive Integrated Transponder (PIT) tag (Mini HPT8 PIT Tag, Biomark, Inc.—Merck Animal Health, Aqua, Boise, ID) to account for recaptures. We injected PIT tags at ~75% of SVL subcutaneously or into the coelomic cavity depending on the snake’s size. Swabs and feces were stored in individual microcentrifuge tubes and placed immediately on ice after collection. Samples were frozen at −20 °C within 3 h of collection and transported on ice to the laboratory, where they were stored at −80 °C until processing. Biosecurity in the field was maintained by donning clean, unused nitrile gloves for each snake handled. Sampling equipment (e.g., tape measure, sexing probes, scale, buckets) was disinfected between individual snakes, and clothing and boots were disinfected between sites using a 5% bleach solution with a minimum contact time of 2 min (71). Any snake carcasses encountered in the field were immediately stored on ice and frozen at −20 °C within 8 h until postmortem examination. All animal sampling was permitted by state and federal agencies (permit numbers provided above) and approved by the University of Georgia Institutional Animal Care and Use Committee (A2020 11-010-Y2-A3 through A2024 02-014-Y1-A0).

### 2.2 Postmortem examination

Most carcasses underwent full postmortem examination, including gross and histopathologic evaluation. In addition, paired tissue samples from all organ systems were collected when available (i.e., not scavenged or too decomposed). One tissue set was stored fresh-frozen at −80 °C, and the second set was fixed in 10% neutral-buffered formalin for histopathology. The latter set was transferred to 70% ethanol within 24–48 h to minimize formalin cross-linkage formation. However, early in the study, postmortem evaluation of a subset of snake carcasses found dead on roads was limited to gross examination and collection of fresh tissue samples. Postmortem swabs and fecal samples were collected from all carcasses and followed the same collection protocol as for live snakes (described above).

For histopathology, tissues routinely examined included skin, head (skin, brain, bone, cartilage, skeletal muscle, glands, teeth, oral mucosa, nasal sinuses, cranial trachea/esophagus), and internal organs (heart, lung, liver, ova/testes, kidneys, stomach, small intestine, large intestine, splenopancreas). We also evaluated tail cross sections (e.g., skin, spinal cord, bone, bone marrow, skeletal muscle, adipose, hemipenes) for most cases. Trimmed tissues were embedded in paraffin and routinely processed for histopathology, including sectioning at 4 μm thick and staining with hematoxylin and eosin, at the University of Georgia College of Veterinary Medicine Histology Laboratory (an American Association of Veterinary Laboratory Diagnosticians-accredited laboratory). Tissues with histologic lesions associated with fungal elements (primarily skin) underwent Grocott’s methenamine silver staining to highlight fungi. All slides were evaluated by a board-certified veterinary pathologist.

### 2.3 Sample analysis

We extracted nucleic acids from swab, fecal, heparinized blood, and tissue (lung and skin if postmortem) samples using the Qiagen DNeasy Blood and Tissue Kit (Qiagen Inc., Valencia, CA). Choanal and cloacal swab samples, heparinized blood, and tissue samples were extracted following the manufacturer’s recommendations. For full-body skin swab extractions, we included an additional incubation step at 37 °C for 1 h with 12.5 units of lyticase per sample (72). Fecal samples were extracted using a modified protocol targeting *Ro* that included PBS wash, freeze, and boil steps (73). When sufficient fecal material remained after extraction, feces were further examined directly via wet-mount microscopy for pentastome ova. We quantified nucleic acid concentrations in all swab extracts (RNA for choanal swabs and DNA for all swabs) using fluorometric quantification (ng/μL; Qubit 4 Fluorometer, Thermo Fisher Scientific, Waltham, MA).

Full-body skin swab (all snakes) and skin sample (carcasses only) extracts were subjected to a previously validated quantitative PCR (qPCR) assay to detect *Oo* DNA (Table 3) (74). We tested samples in triplicate along with an *Oo* gBlock standard dilution curve (Pisces Molecular LLC, Boulder, CO), a DNA extraction negative control, and a non-template control (sterile water) on Bio-Rad CFX96 and Bio-Rad CFX Opus 96 real-time PCR systems (Bio-Rad Laboratories, Inc., Boulder, CO). We considered samples positive if at least two of the three replicates had a mean cycle threshold (Ct) value lower than that of the lowest standard dilution (2.2 molecules/μL) on the same plate. For full-body skin swabs, the mean number of *Oo* DNA copies per sample across replicates was divided by the total DNA concentration measured by spectrophotometry in ng/μL, yielding a standardized number of *Oo* DNA copies per ng DNA extract.

**Table 3:** Primer sets, probes, and cycle parameters for polymerase chain reaction protocols used for pathogen detection in samples from free-ranging snakes in the southeastern United States from 2021 to 2024.

Pathogen	Gene target	Sequences (5–3′)	Cycle parameters	Source
*Cryptosporidium* spp.	18S rRNA	Primary: ACCTATCAGCTTTAGACGGTAGGGTAT TTCTCATAAGGTGCTGAAGGAGTAAGG Secondary: ACAGGGAGGTAGTGACAAGAAATAACA AAGGAGTAAGGAACAACCTCCA	94 °C for 3 min, 39 cycles of 94 °C for 45 s, 56 °C for 45 s, and 72 °C for 1 min, followed by a final extension at 72 °C for 7 min	Silva et al. (78)
*Hepatozoon* spp.	18S rRNA	GTTTCTGACCTATCAGCTTTCGACG CAAATCTAAGAATTTCACCTCTGAC	94 °C for 3 min, 35 cycles of 94 °C for 30 s, 60 °C for 30 s, and 72 °C for 1 min, followed by a final extension at 72 °C for 10 min	Ujvari et al. (80)
*Mycoplasma* spp.	IGS	ACACCATGGGAGYTGGTAAT CTCCWTCGACTTYCAGACCCAAGGCAT	95 °C for 10 min, followed by 35 cycles of 95 °C for 10 s, 60 °C for 30 s, and 72 °C for 30 s, followed by a final extension at 72 °C for 7 min	Rebelo et al. (76)
*Mycoplasma* spp.	16S rRNA	GTGGGAGCAAACAGGATTAGATACCCT TGCACCATCTGTCACTCTGTTAACCTC	94 °C for 2 min, followed by 40 cycles of 94 °C for 30 s, 70 °C for 20 s, and 72 °C for 20 s, followed by a final extension at 72 °C for 7 min	van Kuppeveld et al. (77)
*Ophidiomyces ophidiicola*	ITS1	TGTTTCTGTCTCGCTCGAAGAC AGGTCAAACCGGAAAGAATGG Probe: CGATCGGGCGCCCGTCGTC	50 °C for 2 min, 95 °C for 10 min, 40 cycles of 95 °C for 15 s, and 60 °C for 60 s, followed by a final extension at 72 °C for 10 min	Allender et al. (74)
*Raillietiella orientalis*	COI	GCCTTCTCCATATTACTCCTC CGTATGTTGATGATTGTGGTAGTG	94 °C for 3 min, 35 cycles of 94 °C for 30 s, 56 °C for 30 s, and 72 °C for 60 s, followed by a final extension at 72 °C for 7 min	Palmisano et al. (73)
*Salmonella* spp.	*invA*	GTGAAATTATCGCCACGTTCGGGCAA TCATCGCACCGTCAAAGGAACC	95 °C for 1 min, 38 cycles of 95 °C for 30 s, 64 °C for 30 s, and 72 °C for 30 s, followed by a final extension at 72 °C for 4 min	Upadhyay et al. (79)
Serpentoviruses	RdRp	GAGGACTCCACAARCCAGTCAC RCTRCGGTCGCATTTCGTRTARTC	50 °C for 10 min, 94 °C for 2 min, 40 cycles of 94 °C for 30 s; 42 °C for 30 s, and 72 °C for 30 s, followed by a final extension at 72 °C for 7 min	Hoon-Hanks et al. (75)

rRNA, ribosomal RNA; IGS, intergenic spacer; ITS1, internal transcribed spacer 1; COI, cytochrome c oxidase subunit I; *invA*, invasion protein; RdRp, RNA-dependent RNA polymerase.

Choanal swab extracts were tested for serpentoviruses targeting the RNA-dependent RNA polymerase gene (RdRp) using a conventional reverse transcriptase PCR (Table 3) (75). We also tested choanal swab extracts for *Mycoplasma* spp. targeting the 16S–23S bacterial intergenic spacer (IGS) region via conventional PCR (Table 3) (76). A subset of samples with *Mycoplasma* spp. DNA detected with IGS analysis was further tested targeting the 16S ribosomal RNA (rRNA) gene via conventional PCR (Table 3) (77). Cloacal swabs and fecal extracts were tested for *Salmonella* spp. (gene target: invasion protein [*invA*]), *Cryptosporidium* spp. (gene target: 18S rRNA, nested PCR), and *Raillietiella orientalis* (gene target: cytochrome c oxidase subunit I [COI]) using conventional PCR assays (Table 3) (73, 78, 79). DNA extracts from blood (from live snakes) and lung samples (from carcasses) were tested for *Hepatozoon* spp. (gene target: 18S rRNA) using a conventional PCR assay (Table 3) (80). In addition, blood films were examined via light microscopy for evidence of intraerythrocytic gamonts characteristic of *Hepatozoon* spp. (81, 82).

All conventional PCR assays except those for serpentovirus (i.e., *Mycoplasma* spp., *Salmonella* spp., *Cryptosporidium* spp., *Raillietiella orientalis*, *Hepatozoon* spp.) were run using one of three thermocyclers depending on availability (DNAEngine^®^, Bio-Rad Laboratories, Inc., Hercules, CA; PTC-200, MJ Research Inc., Reno, NV; Mastercycler, Eppendorf SE, Hamburg, Germany). The master mix for all conventional PCR assays (excluding serpentovirus assays) consisted of a 25 μL reaction volume with 5 μL of template DNA, 0.5 μL of 50 μM forward and reverse primers, 11 μL of molecular grade water, 5 μL of Green GoTaq^®^ Flexi Buffer (5×), 0.25 μL of dNTPs (80 mM/dNTP), 2.5 μL of MgCl_2_ (25 mM), and 0.25 μL of GoTaq^®^ Flexi DNA Polymerase (Promega Corporation, Madison, WI). The serpentovirus PCR assay was performed on the ProFlex™ PCR System (Applied Biosystems, Inc., Waltham, MA). The master mix for the serpentovirus PCR assay consisted of a 50 μL reaction volume with 3 μL of template RNA, 4 μL of 10 μM forward and reverse primers, 11.5 μL of molecular grade water, 25 μL of 2× PCRBIO 1-Step Go Mix, and 2.5 μL of 20× RTase Go with RNase inhibitor (PCRBIO 1-Step Go RT-PCR Kit, PCR Biosystems Limited, London, United Kingdom). All primer sets and thermocycler conditions for each PCR reaction are provided in Table 3. Bidirectional Sanger sequencing was performed on all conventional PCR products at the appropriate base pair length through Genewiz (Genewiz from Azenta Life Sciences, South Plainfield, NJ) and sequences were analyzed in Geneious Prime^®^ 2025.1.1 (83).

### 2.4 Statistical analysis

Spatiotemporal and individual predictors for detection of each pathogen were analyzed using binomial generalized linear mixed-effects models [GLMM; R package lme4, function glmer (84)]. Fixed effects included day of year (DOY) of capture, site (two refuge sites and one opportunistic site; inclusion of site in analyses was dependent on detection distribution), their interaction (DOY * site), and recapture status (new capture/recapture), with snake species as a random effect. Physical examination predictors for detection of each pathogen were analyzed using binomial GLMMs with nutritional condition score (ordinal but analyzed as continuous), clinical signs consistent with respiratory disease (presence/absence), recapture status, and sex (male/female/unknown) as fixed effects, with site (only included in models for which detection distribution justified inclusion) and species as random effects. We defined day 1 of DOY as the first day of winter (December 21), which was z-transformed for all models. Species for which fewer than 10 individuals were sampled were collectively classified as “other.”

We analyzed lesion severity score (ordinal but analyzed as continuous) for snakes with *Oo* detected based on DOY, coinfection presence (present/absent), standardized *Oo* DNA concentration (*Oo* DNA copies per ng DNA extract; z-transformed), site, and recapture status as fixed effects, with species as a random effect, using a Poisson GLMM. Multinomial logistic regression modeling [MLRM; R package nnet, function multinom (85)] was used to predict ophidiomycosis case classification based on species, site, DOY, recapture status, and coinfection presence. Ophidiomycosis case classifications were assigned following the criteria outlined by Baker et al. (14): negative—no skin lesions and *Oo* not detected; present—no skin lesions and *Oo* detected; possible—skin lesions present and *Oo* not detected; apparent—skin lesions present and *Oo* detected. Snakes that fit the category of “negative” were used as the model reference. MLRM results for each species were assessed through *post hoc* pairwise comparisons with Tukey’s *p*-value adjustment [R package and function emmeans (86)].

Odd ratios (OR; for binomial and multinomial models) and rate ratios (RR; for Poisson models) were reported for significant predictors. All models were assessed for collinearity using variance inflation factors [VIF; R package car, function vif (87)], and variables with VIF >2 were not included. Overdispersion for GLMMs was assessed based on Pearson residuals. Models were designed as outlined above to test specific biological hypotheses. Unweighted Cohen’s kappa agreement tests were used to evaluate agreement of *Cryptosporidium* spp., *Salmonella* spp., and *Ro* detection between cloacal swabs and feces, and *Hepatozoon* detection between blood PCR and blood film microscopy (performed on data excluding recapture snakes). Kappa values >0.8 were considered excellent, 0.6–0.8 were considered good, 0.4–0.6 were considered moderate, and <0.4 were considered poor. Pathogen detection prevalence was calculated excluding recaptures. We performed all data analyses in R version 4.4.3 (R Core Team 2025 (88)), with statistical significance at *α* = 0.05 (*p* ≤ 0.05).

## 3 Results

### 3.1 Study animals

We sampled a total of 509 individual snakes, including 49 recaptures, representing 29 species from 21 counties within the southeastern United States. During the monthly refuge surveys from May 2022 to May 2024, we sampled 289 (*n* = 12 found dead, *n* = 5 recaptures found dead) snakes from Volusia County, FL, and 144 (*n* = 11 found dead) snakes from Jasper County, SC. Opportunistically collected samples included 43 live snakes from Athens-Clarke County, GA, and 33 diagnostic carcass submissions to Southeastern Cooperative Wildlife Disease Study (SCWDS) [Florida: *n* = 5, Georgia: *n* = 15 (8 from Athens-Clarke County), Louisiana: *n* = 1, South Carolina: *n* = 11 (2 from Jasper County), and Tennessee: *n* = 1]. The sex distribution of snakes sampled was nearly equal, with 276 female, 220 male, and 13 snakes for which sex was not determined, either due to small body size or poor carcass condition (Table 4). The age profile included 147 juveniles and 362 adults (Table 4). A complete list of species and their respective sample sizes is provided in Table 5.

**Table 4:** Sample size and pathogen detection prevalence by age group, sex, and counties with >10 snakes sampled across the southeastern United States from 2021 to 2024, excluding recaptures.

Pathogen		*Cryptosporidium* spp.		*Hepatozoon* spp.		*Mycoplasma* spp.		*Ophidiomyces ophidiicola*		*Raillietiella orientalis*		*Salmonella enterica*	
	*n*^t^	+/*n*	% (CI)	+/*n*	% (CI)	+/*n*	% (CI)	+/*n*	% (CI)	+/*n*	% (CI)	+/*n*	% (CI)
Age													
Juvenile	147	$\frac{0}{142}$	0 (0–2.6)	$\frac{19}{88}$	21.6% (14.3–31.3)	$\frac{5}{131}$	3.8% (1.6–8.6)	$\frac{10}{147}$	6.8% (3.7–12.1)	$\frac{0}{89}$	0 (0–4.1)	$\frac{78}{142}$	54.9% (46.7–62.9)
Adult	362	$\frac{10}{347}$	2.9% (1.6–5.2)	$\frac{186}{296}$	62.8% (57.2–68.1)	$\frac{73}{314}$	23.2% (18.9–28.2)	$\frac{72}{361}$	19.9% (16.1–24.4)	$\frac{37}{203}$	18.2% (13.5–24.1)	$\frac{228}{347}$	65.7% (60.6–70.5)
Sex													
Female	276	$\frac{6}{269}$	2.2% (1.0–4.8)	$\frac{114}{213}$	53.5% (46.8–60.1)	$\frac{41}{236}$	17.4% (13.1–22.7)	$\frac{49}{275}$	17.8% (13.7–22.8)	$\frac{18}{162}$	11.1% (7.1–16.9)	$\frac{170}{269}$	63.2% (57.3–68.7)
Male	220	$\frac{4}{216}$	1.9% (0.7–4.7)	$\frac{90}{170}$	52.9% (45.5–60.3)	$\frac{36}{205}$	17.6% (13.0–23.4)	$\frac{31}{220}$	14.9% (10.1–19.3)	$\frac{19}{129}$	14.7% (9.6–21.9)	$\frac{133}{216}$	61.6% (54.9–67.8)
Unknown	13	$\frac{0}{4}$	0 (0–49.0)	$\frac{1}{1}$	100% (20.7–100)	$\frac{1}{4}$	25.0% (4.6–69.9)	$\frac{2}{13}$	15.4% (4.3–42.2)	$\frac{0}{1}$	0 (0–79.3)	$\frac{3}{4}$	75.0% (30.1–95.4)
County													
Athens-Clarke, Georgia	51	$\frac{0}{37}$	0 (0–9.4)	$\frac{14}{28}$	50.0% (32.6–67.4)	$\frac{2}{38}$	5.3% (1.5–17.3)	$\frac{14}{50}$	28.0% (17.5–41.7)	NA	NA	$\frac{27}{37}$	73.0% (57.0–84.6)
Jasper, South Carolina	146	$\frac{2}{145}$	1.4% (0.4–4.9)	$\frac{61}{116}$	52.6% (43.6–61.4)	$\frac{1}{132}$	0.8% (0.1–4.2)	$\frac{30}{146}$	20.5% (14.8–27.8)	NA	NA	$\frac{94}{145}$	64.8% (56.8–72.1)
Volusia, Florida	289	$\frac{7}{286}$	2.4% (1.2–5.0)	$\frac{122}{220}$	55.5% (48.8–61.9)	$\frac{72}{255}$	28.2% (23.1–34.1)	$\frac{33}{289}$	11.4% (8.2–15.6)	$\frac{35}{287}$	12.2% (8.9–16.5)	$\frac{172}{286}$	60.1% (54.4–65.6)
Overall	509	$\frac{10}{489}$	2.0% (1.1–3.7)	$\frac{205}{384}$	53.4% (48.4–58.3)	$\frac{78}{445}$	17.5% (14.3–21.3)	$\frac{82}{508}$	16.1% (13.2–19.6)	$\frac{37}{292}$	12.7% (9.3–17.0)	$\frac{306}{489}$	62.6% (58.2–66.8)

Detection prevalence is shown for each pathogen with 95% confidence intervals calculated using the Wilson score interval (120, 121) in parentheses. Data for *Raillietiella orientalis* only include snakes from Florida. *n*^t^, total study sample size; +/*n*, number of detections/number of samples tested; %, detection prevalence; CI, 95% confidence interval; NA, not available.

**Table 5:** Sample size and pathogen detection prevalence by snake species sampled across the southeastern United States from 2021 to 2024, excluding recaptures.

Species	Common name		*Cryptosporidium* spp.		*Hepatozoon* spp.		*Mycoplasma* spp.		*Ophidiomyces ophidiicola*		*Salmonella enterica*	
		*n*^t^	+/*n*	% (CI)	+/*n*	% (CI)	+/*n*	% (CI)	+/*n*	% (CI)	+/*n*	% (CI)
*Agkistrodon contortrix*	Eastern copperhead	6	$\frac{0}{6}$	0 (0–39.0)	$\frac{4}{6}$	66.7% (30–90.3)	$\frac{0}{1}$	0 (0–79.3)	$\frac{2}{6}$	33.3% (9.7–70.0)	$\frac{5}{6}$	83.3% (43.6–97.0)
*A. piscivorus*	Northern cottonmouth	6	$\frac{0}{6}$	0 (0–39.0)	$\frac{4}{6}$	66.7% (30.0–90.3)	$\frac{0}{1}$	0 (0–79.3)	$\frac{3}{6}$	50.0% (18.8–81.2)	$\frac{4}{6}$	66.7% (30.0–90.3)
*Carphophis amoenus*	Common worm snake	3	$\frac{0}{2}$	0 (0–65.8)	$\frac{0}{1}$	0 (0–79.3)	$\frac{0}{2}$	0 (0–65.8)	$\frac{1}{3}$	33.3% (6.1–79.2)	$\frac{0}{2}$	0 (0–65.8)
*Cemophora coccinea*	Common scarlet snake	2	$\frac{0}{2}$	0 (0–65.8)	$\frac{0}{1}$	0 (0–79.3)	$\frac{0}{2}$	0 (0–65.8)	$\frac{0}{2}$	0 (0–65.8)	$\frac{2}{2}$	100% (34.2–100)
***Coluber constrictor***	**Black racer**	**56**	$\frac{1}{54}$	**1.9% (0.3–9.8)**	$\frac{45}{49}$	**91.8% (80.8–96.8)**	$\frac{10}{55}$	**18.2% (10.2–30.3)**	$\frac{9}{56}$	**16.1% (8.7–27.8)**	$\frac{53}{54}$	**98.1% (90.2–99.7)**
*Crotalus horridus*	Timber rattlesnake	4	$\frac{0}{4}$	0 (0–49.0)	$\frac{1}{4}$	25.0% (4.6–69.9)	$\frac{0}{1}$	0 (0–79.3)	$\frac{1}{4}$	25.0% (4.6–69.9)	$\frac{4}{4}$	100% (51.0–100)
***Diadophis punctatus***	**Ring-necked snake**	**36**	$\frac{2}{33}$	**6.1% (1.7–19.6)**	$\frac{0}{4}$	0 (0–49.0)	$\frac{12}{35}$	**34.3% (20.8–50.8)**	$\frac{3}{36}$	**8.3% (2.9–21.8)**	$\frac{19}{33}$	**57.6% (40.8–72.8)**
*Drymarchon couperi*	Eastern indigo snake	1	$\frac{1}{1}$	100% (20.7–100)	0	NA	$\frac{0}{1}$	0 (0–79.3)	$\frac{1}{1}$	100% (20.7–100)	$\frac{0}{1}$	0 (0–79.3)
*Farancia abacura*	Red-bellied mud snake	4	$\frac{0}{4}$	0 (0–49.0)	$\frac{0}{2}$	0 (0–65.8)	$\frac{1}{4}$	25.0% (4.6–69.9)	$\frac{2}{4}$	50.0% (15.0–85.0)	$\frac{1}{4}$	25.0% (4.6–69.9)
*F. erytrogramma*	Rainbow snake	2	$\frac{0}{2}$	0 (0–65.8)	$\frac{0}{2}$	0 (0–65.8)	$\frac{0}{2}$	0 (0–65.8)	$\frac{2}{2}$	100% (34.2–100)	$\frac{2}{2}$	100% (34.2–100)
*Lampropeltis elapsoides*	Scarlet kingsnake	4	$\frac{0}{4}$	0 (0–49.0)	$\frac{1}{1}$	100% (20.7–100)	$\frac{0}{4}$	0 (0–49.0)	$\frac{0}{4}$	0 (0–49.0)	$\frac{4}{4}$	100% (51.0–100)
*L. getula*	Eastern kingsnake	2	$\frac{0}{2}$	0 (0–65.8)	$\frac{2}{2}$	100% (34.2–100)	$\frac{0}{2}$	0 (0–65.8)	$\frac{1}{2}$	50.0% (9.5–90.5)	$\frac{2}{2}$	100% (34.2–100)
***Liodytes alleni***	**Striped crayfish snake**	**12**	$\frac{2}{12}$	**16.7% (4.7–44.8)**	$\frac{0}{10}$	**0 (0–27.8)**	$\frac{3}{12}$	**25.0% (8.9–53.2)**	$\frac{1}{12}$	**8.3% (1.5–35.4)**	$\frac{4}{12}$	**33.3% (13.8–60.9)**
*L. rigida*	Glossy crayfish snake	1	$\frac{0}{1}$	0 (0–79.3)	$\frac{0}{1}$	0 (0–79.3)	$\frac{0}{1}$	0 (0–79.3)	$\frac{0}{1}$	0 (0–79.3)	$\frac{1}{1}$	100% (20.7–100)
*Nerodia cyclopion*	MS green watersnake	1	$\frac{0}{1}$	0 (0–79.3)	$\frac{1}{1}$	100% (20.7–100)	$\frac{0}{1}$	0 (0–79.3)	$\frac{1}{1}$	100% (20.7–100)	$\frac{0}{1}$	0 (0–79.3)
***N. fasciata***	**Banded watersnake**	**141**	$\frac{3}{141}$	**2.1% (0.7–6.1)**	$\frac{70}{130}$	**53.8% (45.3–62.2)**	$\frac{20}{140}$	**14.3% (9.4–21.0)**	$\frac{15}{141}$	**10.6% (6.6–16.8)**	$\frac{62}{141}$	**44.0% (36.0–52.2)**
***N. floridana***	**Florida green watersnake**	**36**	$\frac{0}{35}$	**0 (0–9.9)**	$\frac{4}{35}$	**11.4% (4.5–26.0)**	$\frac{9}{35}$	**25.7% (14.2–42.1)**	$\frac{4}{36}$	**11.1% (4.4–25.3)**	$\frac{9}{35}$	**25.7% (14.2–42.1)**
*N. sipedon*	Northern watersnake	7	$\frac{0}{7}$	0 (0–35.4)	$\frac{7}{7}$	100% (64.6–100)	$\frac{0}{7}$	0 (0–35.4)	$\frac{3}{7}$	42.9% (15.8–75.0)	$\frac{4}{7}$	57.1% (25.0–84.2)
*N. taxispilota*	Brown watersnake	5	$\frac{0}{5}$	0 (0–43.4)	$\frac{0}{5}$	0 (0–43.4)	$\frac{0}{4}$	0 (0–49.0)	$\frac{3}{5}$	60.0% (23.1–88.2)	$\frac{2}{5}$	40.0% (11.8–76.9)
*Opheodrys aestivus*	Rough green snake	5	$\frac{0}{4}$	0 (0–49.0)	$\frac{0}{2}$	0 (0–65.8)	$\frac{1}{5}$	20.0% (3.6–62.4)	$\frac{0}{5}$	0 (0–43.4)	$\frac{3}{4}$	75.0% (30.1–95.4)
***Pantherophis alleghaniensis***	**Eastern rat snake**	**34**	$\frac{0}{27}$	**0 (0–12.5)**	$\frac{14}{26}$	**53.8% (35.5–71.2)**	$\frac{1}{25}$	**4.0% (0.7–19.5)**	$\frac{10}{33}$	**30.3% (17.4–47.3)**	$\frac{26}{27}$	**96.3% (81.7–99.3)**
*P. guttatus*	Corn snake	4	$\frac{0}{4}$	0 (0–49.0)	$\frac{1}{3}$	33.3% (6.1–79.2)	$\frac{0}{3}$	0 (0–56.1)	$\frac{2}{4}$	50.0% (15.0–85.0)	$\frac{4}{4}$	100% (51.0–100)
*Pituophis melanoleucus*	Eastern pine snake	2	$\frac{0}{2}$	0 (0–65.8)	$\frac{0}{2}$	0 (0–65.8)	$\frac{1}{2}$	50.0% (9.5–90.5)	$\frac{0}{2}$	0 (0–65.8)	$\frac{1}{2}$	50.0% (9.5–90.5)
***Seminatrix pygaea***	**Black swamp snake**	**15**	$\frac{0}{14}$	**0 (0–21.5)**	$\frac{4}{8}$	50.0% (21.5–78.5)	$\frac{0}{15}$	**0 (0–20.4)**	$\frac{1}{15}$	**6.7% (1.2–29.8)**	$\frac{3}{14}$	**21.4% (7.6–47.6)**
***Sistrurus miliarius***	**Pygmy rattlesnake**	**34**	$\frac{0}{33}$	**0 (0–10.4)**	$\frac{20}{26}$	**76.9% (57.9–89.0)**	$\frac{0}{3}$	0 (0–56.1)	$\frac{12}{34}$	**35.3% (21.5–52.1)**	$\frac{32}{33}$	**97.0% (84.7–99.5)**
*Storeria dekayi*	Dekay’s brown snake	3	$\frac{0}{3}$	0 (0–56.1)	$\frac{0}{2}$	0 (0–65.8)	$\frac{0}{3}$	0 (0–56.1)	$\frac{0}{3}$	0 (0–56.1)	$\frac{1}{3}$	33.3% (6.1–79.2)
*S. occipitomaculata*	Red-bellied snake	8	$\frac{0}{5}$	0 (0–43.4)	0	NA	$\frac{0}{5}$	0 (0–43.4)	$\frac{0}{8}$	0 (0–32.4)	$\frac{2}{5}$	40.0% (11.8–76.9)
***Thamnophis saurita***	**Eastern ribbon snake**	**55**	$\frac{0}{55}$	**0 (0–6.5)**	$\frac{17}{32}$	**53.1% (36.4–69.1)**	$\frac{14}{55}$	**25.5% (15.8–38.3)**	$\frac{1}{55}$	**1.8% (0.3–9.6)**	$\frac{41}{55}$	**74.5% (61.7–84.2)**
***T. sirtalis***	**Common garter snake**	**20**	$\frac{1}{20}$	**5.0% (0.9–23.6)**	$\frac{10}{16}$	**62.5% (38.6–81.5)**	$\frac{6}{19}$	**31.6% (15.4–54.0)**	$\frac{4}{20}$	**20.0% (8.1–41.6)**	$\frac{15}{20}$	**75.0% (53.1–88.8)**
	Overall	509	$\frac{10}{489}$	2.0% (1.1–3.7)	$\frac{205}{384}$	53.4% (48.4–58.3)	$\frac{78}{445}$	17.5% (14.3–21.3)	$\frac{82}{508}$	16.1% (13.2–19.6)	$\frac{306}{489}$	62.6% (58.2–66.8)

Detection prevalence is shown for each pathogen with 95% confidence intervals calculated using the Wilson score interval (120, 121) in parentheses. Bolded species indicate those included individually (*n* > 10) in model analyses; all other species were classified as “other.” *n*^t^, total study sample size; +/*n*, number of detections/number of samples tested; %, detection prevalence; CI, 95% confidence interval; MS, Mississippi; NA, not available.

### 3.2 Pathogen detection prevalence, excluding recaptures

No pathogens were detected in 19.3% of snakes [96/498; 95% confidence interval (CI): 16.1–23.0%], of which 44.8% (43/96) were classified as adults and 55.2% (53/96) as juveniles. We detected *Oo* in 16.1% of snakes (82/508; 95% CI: 13.2–19.6%; Table 4), including detections from full-body skin swabs and/or skin extracts. Overall *Cryptosporidium* spp. detection prevalence among all tested snakes for which at least one sample type (cloacal swabs, feces, and/or tissue extracts) was available was 2.0% (10/489; 95% CI: 1.1–3.7%; Table 4). Sequence [550 base pairs (bp)] analysis indicated that 9/10 had 99–100% identity to *C. serpentis* (GenBank IDs: PZ281950-PZ281955, PZ281957-PZ281961). The sequence that did not match *C. serpentis* was from a banded watersnake (*Nerodia fasciata*) in Jasper County, SC, and the 547-bp amplicon (GenBank ID: PZ281956) had a 90.7% identity to *Cryptosporidium* sp. from a Japanese eel fry (*Anguilla japonica*; GenBank ID: JX436322). Agreement between cloacal swab and fecal detection of *Cryptosporidium* spp. was poor (*K* = 0.27; −0.17–0.72) among snakes with paired samples (*n* = 219), with prevalence of 1.2% (6/485; 95% CI: 0.6–2.7%) in cloacal swabs and 1.8% (4/222; 95% CI: 0.7–4.5%) in feces. Of snakes with paired sample types tested, *Cryptosporidium* spp. were detected in one snake via both methods, in three snakes via feces only, and in two snakes via cloacal swab only. Based on the low detection prevalence, *Cryptosporidium* spp. were excluded from individual pathogen analysis.

We detected *Salmonella enterica* in 62.6% of snakes (306/489; 95% CI: 58.2–66.8%; Table 4), including detections from cloacal swabs and/or fecal extracts. All sequences (260 bp, GenBank IDs: PZ289793-PZ289798) matched *S. enterica* with 99–100% identity, although the analysis lacked the discriminatory power to determine subspecies or serovar. We detected *S. enterica* in 60.0% (291/485; 55.6–64.3%) of cloacal swabs and 32.3% (72/223; 95% CI: 26.5–38.7%) of fecal samples. There was poor agreement (*K* = 0.19; 0.09–0.29) of *S. enterica* detection between cloacal swabs and feces among snakes with paired samples (*n* = 219). Among these, *S. enterica* was detected in 25.6% (56/219) of snakes by both methods, in 6.8% (15/219) by feces only, and in 37.4% (82/219) by cloacal swab only.

Overall *Hepatozoon* spp. detection prevalence from blood, tissue extracts, and/or microscopic blood film examination was 53.4% of snakes (205/384; 95% CI: 48.4–58.3%; Table 4). We detected *Hepatozoon* spp. in 48.5% (175/361; 95% CI: 43.4–53.6%) of blood and/or tissue extracts via PCR and in 49.9% (167/335; 95% CI: 44.5–55.2%) of blood films via light microscopy. Agreement between *Hepatozoon* spp. detection via PCR and microscopy was good (*K* = 0.74; 0.66–0.81). Among snakes evaluated by both PCR and microscopy (*n* = 312), *Hepatozoon* spp. was detected in 43.9% (137/312) of snakes via both methods, 6.1% (19/312) snakes via microscopy only, and 7.1% (22/312) snakes via PCR only. Sequence analysis revealed five unique clusters. Most sequences (600 bp) belonged to Cluster A (*n* = 116, GenBank ID: PZ281992), which had 100% identity to *Hepatozoon* sp. YLW-2014 (GenBank ID: KF939622; Supplementary Figure 1). Cluster B (*n* = 23, GenBank ID: PZ281991) had 100% identity to *Hepatozoon cuestensis* (GenBank ID: ON237459; Supplementary Figure 1). Cluster C (*n* = 23, GenBank ID: PZ281989) had 100% identity to *Hepatozoon* cf. *sipedon* (GenBank ID: PP795209; Supplementary Figure 1). Cluster D (*n* = 8, GenBank ID: PZ281990) had a 99.66% identity to *Hepatozoon* sp. isolate FP3 (GenBank ID: MT561455; Supplementary Figure 1). Cluster E was limited to one sequence from a canebrake rattlesnake (*Crotalus horridus*, GenBank ID: PZ281993) from the South Carolina site with a 99.66% identity to *Hepatozoon ameivae* (GenBank ID: MN833641; Supplementary Figure 1).

*Mycoplasma* spp. detection prevalence, including choanal swabs and/or tissue extracts, was 17.5% of snakes (78/445; 95% CI: 14.3–21.3%; Table 4). Sequence analysis using the IGS gene target (722 bp) revealed five unique clusters. Most sequences belonged to Cluster A (*n* = 56, GenBank ID: PZ288836), with fewer in Clusters B (*n* = 4, GenBank ID: PZ288834), C (*n* = 6, GenBank ID: PZ288837), D (*n* = 3, GenBank ID: PZ288835), and E (*n* = 8, GenBank ID: PZ288833; Supplementary Figure 2). Representatives from each cluster, analyzed using the 16S gene target (167 bp, GenBank IDs: PZ282827-PZ282838), all matched the same *Mycoplasma* sp. from a captive western hognose snake (*Heterodon nasicus*; GenBank ID: PQ452199) with a 98.6–100% identity (Supplementary Figure 3). No serpentoviruses were detected in choanal swab and/or lung extracts (*n* = 447, 95% CI: 0–0.9%); therefore, serpentovirus results were excluded from further analysis.

We did not detect *Ro* in fecal or swab extracts, nor pentastome ova on fecal wet mounts, in snakes caught outside of Florida; therefore, *Ro* prevalence calculation and analysis were limited to snakes from Florida. Overall detection prevalence of *Ro* was 12.7% of snakes (37/292; 95% CI: 9.3–17.0%; Table 6) in cloacal swabs, fecal samples, and/or adult pentastomes recovered during postmortem evaluation. All sequences (150 bp, GenBank ID: PZ288801) matched *Raillietiella orientalis* with 99–100% identity. Detection prevalence was lower in cloacal swab samples (2.8%; 8/288; 95% CI: 1.4–5.4%) *versus* fecal samples (21.9%; 32/146; 95% CI: 16.0–29.3%; Table 6). Agreement was poor (*K* = 0.17; 0.01–0.33) for *Ro* detection between cloacal swabs and feces in snakes with paired sample collection (*n* = 142). Among these, *Ro* was detected in 2.8% (4/142) of snakes via both methods, 19.0% (27/142) of snakes via feces only, and 0.7% (1/142) of snakes via cloacal swab only. Fecal wet-mount microscopy revealed pentastome ova in 33.6% (42/125; CI: 25.9–42.3%) of fecal samples (Table 6).

**Table 6:** Sample size and detection prevalence of *Raillietiella orientalis* by polymerase chain reaction and pentastome ova observed on fecal wet mounts by snake species, age, and sex of snakes sampled from Florida, United States, from 2022 to 2024, excluding recaptures.

		*Raillietiella orientalis*						Pentastome ova fecal wet-mount microscopy %	
		Overall %		Cloacal swab %			Fecal %		
Species	Common name	+/*n*	% (CI)	+/*n*	% (CI)	+/*n*	% (CI)	+/*n*	% (CI)
*Cemophora coccinea*	Common scarlet snake	$\frac{0}{1}$	0 (0–79.3)	$\frac{0}{1}$	0 (0–79.3)	0	NA	0	NA
***Coluber constrictor***	**Black racer**	$\frac{6}{21}$	**28.6% (13.8–50.0)**	$\frac{1}{21}$	**4.8% (0.8–22.7)**	$\frac{5}{14}$	**35.7% (16.3–61.2)**	$\frac{8}{13}$	**61.5% (35.5–82.3)**
***Diadophis punctatus***	**Ring-necked snake**	$\frac{0}{20}$	**0 (0–16.1)**	$\frac{0}{18}$	**0 (0–17.6)**	$\frac{0}{12}$	**0 (0–24.2)**	$\frac{0}{5}$	**0 (0–43.4)**
*Drymarchon couperi*	Eastern indigo snake	$\frac{0}{1}$	0 (0–79.3)	$\frac{0}{1}$	0 (0–79.3)	0	NA	0	NA
*Farancia abacura*	Red-bellied mud snake	$\frac{0}{4}$	0 (0–49.0)	$\frac{0}{4}$	0 (0–49.0)	$\frac{0}{1}$	0 (0–79.3)	$\frac{0}{1}$	0 (0–79.3)
*Lampropeltis elapsoides*	Scarlet kingsnake	$\frac{0}{4}$	0 (0–49.0)	$\frac{0}{4}$	0 (0–49.0)	$\frac{0}{1}$	0 (0–79.3)	$\frac{0}{1}$	0 (0–79.3)
***Liodytes alleni***	**Striped crayfish snake**	$\frac{0}{12}$	**0 (0–24.2)**	$\frac{0}{12}$	**0 (0–24.2)**	$\frac{0}{10}$	**0 (0–27.8)**	$\frac{0}{9}$	**0 (0–29.9)**
***Nerodia fasciata***	**Banded watersnake**	$\frac{7}{84}$	**8.3% (4.1–16.2)**	$\frac{1}{84}$	**1.2% (0.2–6.4)**	$\frac{6}{31}$	**19.4% (9.2–36.3)**	$\frac{9}{29}$	**31.0% (17.3–49.2)**
***N. floridana***	**Florida green watersnake**	$\frac{0}{32}$	**0 (0–10.7)**	$\frac{0}{32}$	**0 (0–10.7)**	$\frac{0}{16}$	**0 (0–19.4)**	$\frac{2}{16}$	**12.5% (3.5–36.0)**
*N. taxispilota*	Brown watersnake	$\frac{0}{1}$	0 (0–79.3)	$\frac{0}{1}$	0 (0–79.3)	$\frac{0}{1}$	0 (0–79.3)	$\frac{0}{1}$	0 (0–79.3)
*Pantherophis alleghaniensis*	Eastern rat snake	$\frac{0}{6}$	0 (0–39.0)	$\frac{0}{6}$	0 (0–39.0)	$\frac{0}{4}$	0 (0–49.0)	$\frac{0}{3}$	0 (0–56.1)
*P. guttatus*	Corn snake	$\frac{1}{1}$	100% (20.7–100)	$\frac{0}{1}$	0 (0–79.3)	$\frac{1}{1}$	100% (20.7–100)	$\frac{1}{1}$	100% (20.7–100)
***Seminatrix pygaea***	**Black swamp snake**	$\frac{1}{13}$	**7.7% (1.4–33.3)**	$\frac{1}{13}$	**7.7% (1.4–33.3)**	$\frac{0}{2}$	**0 (0–65.8)**	$\frac{0}{2}$	**0 (0–65.8)**
***Sistrurus miliarius***	**Pygmy rattlesnake**	$\frac{14}{34}$	**41.2% (26.4–57.8)**	$\frac{5}{33}$	**15.2% (6.7–30.9)**	$\frac{13}{18}$	**72.2% (49.1–87.5)**	$\frac{11}{18}$	**61.1% (38.6–79.7)**
***Thamnophis saurita***	**Eastern ribbon snake**	$\frac{3}{42}$	**7.1% (2.5–19.0)**	$\frac{0}{41}$	**0 (0–8.6)**	$\frac{3}{22}$	**13.6% (4.7–33.3)**	$\frac{6}{14}$	**42.9% (21.4–67.4)**
***T. sirtalis***	**Common garter snake**	$\frac{5}{16}$	**31.3% (14.2–55.6)**	$\frac{0}{16}$	**0 (0–19.4)**	$\frac{4}{13}$	**30.8% (12.7–57.6)**	$\frac{5}{12}$	**41.7% (19.3–68.0)**
	Overall	$\frac{37}{292}$	12.7% (9.3–17.0)	$\frac{8}{288}$	2.8% (1.4–5.4)	$\frac{32}{146}$	21.9% (16.0–29.3)	$\frac{42}{125}$	33.6% (25.9–42.3)

Detection prevalence is shown for each pathogen with 95% confidence intervals calculated using the Wilson score interval (120, 121) in parentheses. Bolded species indicate species those analyzed separately in model analyses; all other species were classified as “other.” +/*n*, number of detections/number of samples tested; %, detection prevalence; CI, 95% confidence interval; NA, not available.

Non-target parasite findings during physical examination of live snakes included unidentified scale mites (*n* = 18) and oral trematodes (likely *Reniferidae* species based on morphology; *n* = 72) (89). Mites were only observed on snakes from the refuge sites (13 from the South Carolina site and five from the Florida site) and were predominately observed on black racers (*Coluber constrictor*; *n* = 10), along with one eastern copperhead (*Agkistrodon contortrix*), two ring-necked snakes (*Diadophis punctatus*), two banded watersnakes, two eastern rat snakes (*Pantherophis alleghaniensis*), and one common garter snake (*Thamnophis sirtalis*). Oral trematodes were observed in snakes at both refuge sites (21 from South Carolina, 48 from Florida) and three from opportunistic collection in Georgia. Oral trematodes were predominately observed in banded watersnakes (*n* = 47), as well as in 12 Florida green watersnakes, nine black racers, one northern cottonmouth (*Agkistrodon piscivorus*), one northern watersnake (*Nerodia sipedon*), one eastern rat snake, and one common garter snake.

### 3.3 Coinfection prevalence, excluding recaptures

**Figure 2:**
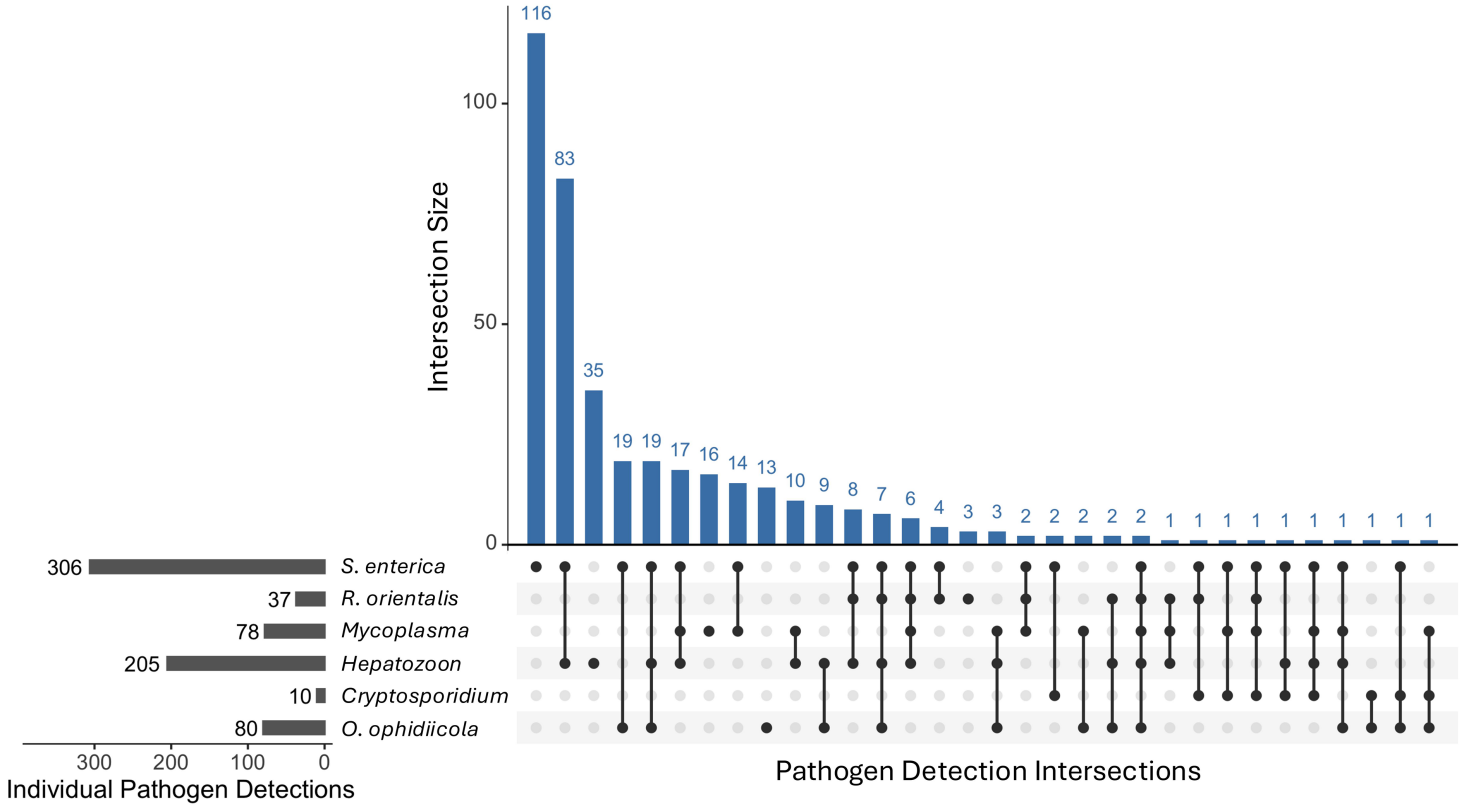
The top bar plot summarizes the number of free-ranging snakes sampled from the southeastern United States from 2021 to 2024 with either a single infection or coinfections detected. The side bar plot represents the single pathogen detection data without the subtraction of coinfections. The plot was created using the UpSetR package (119).

Overall coinfection prevalence was 44.0% of snakes (219/498, 95% CI: 39.7–48.4%) with 27 unique coinfection combinations (Figure 2). Of these, 28.9% of snakes (144/498, 95% CI: 25.1–33.0%) were coinfected with two pathogens, 11.4% of snakes (57/498, 95% CI: 8.9–14.5%) with three, 3.2% of snakes (16/498, 95% CI: 2.0–5.2%) with four, and two snakes had coinfections with five pathogens. The most common coinfection groups were: *Hepatozoon* spp. and *S. enterica* (*n* = 83); *Oo*, *Hepatozoon* spp. and *S. enterica* (*n* = 19); and *Oo* and *S. enterica* (*n* = 19; Figure 2). Coinfections were commonly detected in snakes, with all targeted pathogens being detected (e.g., all snakes infected with *Cryptosporidium* spp. were coinfected with a variety of other pathogens; Figure 2).

### 3.4 Pathogen detection analyses

**Figure 3:**
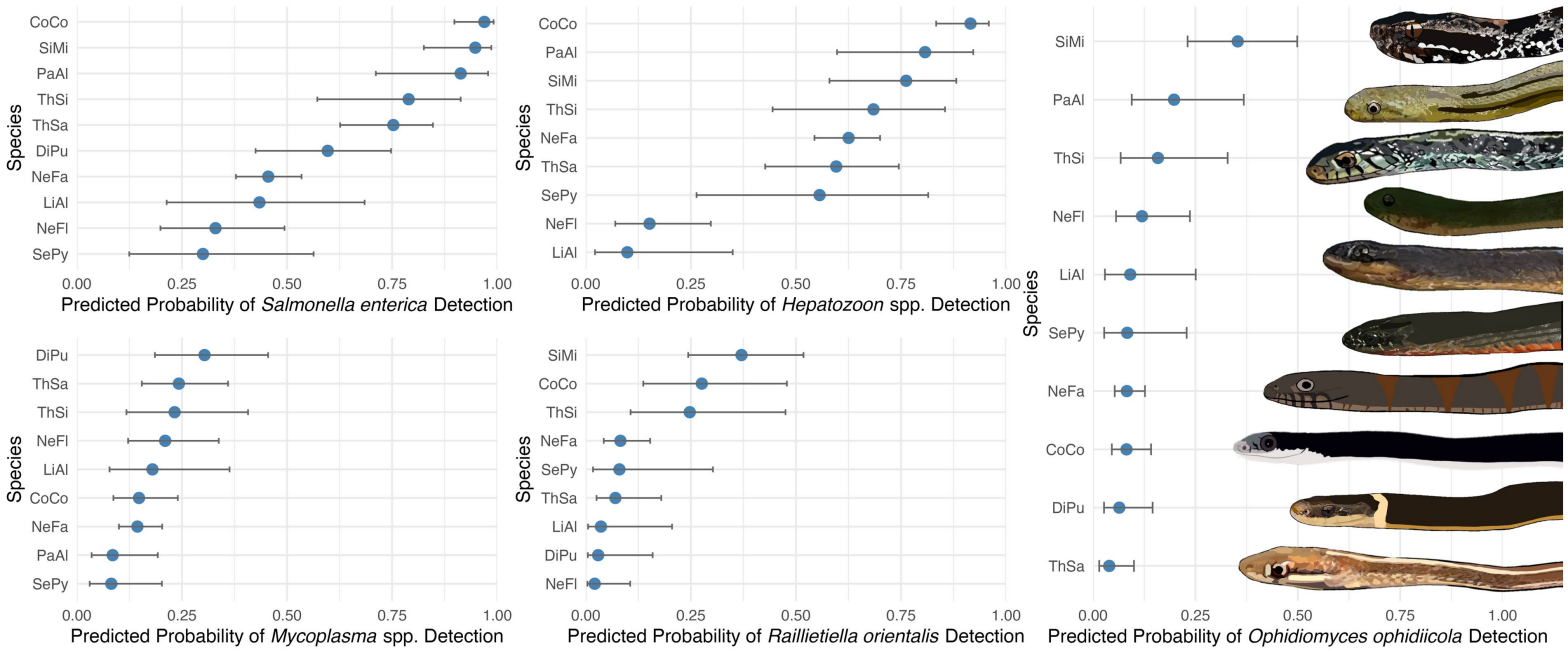
Predicted probability of pathogen detection by snake species using generalized linear mixed models. Free-ranging snake species with greater than 10 samples collected from 2021 to 2024 in the southeastern United States were analyzed individually, whereas those with too few samples were classified as “other.” *Raillietiella orientalis* analysis was limited to snakes from Florida. Species codes: CoCo: black racer (*Coluber constrictor*), DiPu: ring-necked snake (*Diadophis punctatus*), LiAl: striped crayfish snake (*Liodytes alleni*), NeFa: banded watersnake (*Nerodia fasciata*), NeFl: Florida green watersnake (*N. floridana*), PaAl: eastern rat snake (*Pantherophis alleghaniensis*), SePy: black swamp snake (*Seminatrix pygaea*), SiMi: pygmy rattlesnake (*Sistrurus miliarius*), ThSa: eastern ribbon snake (*Thamnophis saurita*), ThSi: common garter snake (*T. sirtalis*).

Host species was strongly predictive of pathogen detection, as summarized in Figure 3. For *Oo* detection, pygmy rattlesnakes (*Sistrurus miliarius*) had the highest predicted detection probability (12/34 with *Oo* detected; OR = 0.5463, SE = 0.306, *p* = 0.0479), whereas eastern ribbon snakes (1/55 with *Oo* detected; OR = 0.0412, SE = 0.505, *p* < 0.0001; *Thamnophis saurita*) and ring-necked snakes (3/36 with *Oo* detected; OR = 0.0685, SE = 0.464, *p* < 0.0001) had the lowest predicted detection probabilities (Table 5 and Figure 3). For *S. enterica* detection, black racers (53/54 with *S. enterica* detected; OR = 31.50, SE = 0.651, *p* < 0.0001) and pygmy rattlesnakes (32/33 with *S. enterica* detected; OR = 18.11, SE = 0.686, *p* < 0.0001) had the highest predicted detection probability with the lowest detection probability in black swamp snakes (3/14; OR = 0.4283, SE = 0.562, *p* = 0.1314; *Seminatrix pygaea*; Table 5 and Figure 3). For *Hepatozoon* spp., the highest detection probability was in black racers (45/49 with *Hepatozoon* spp. detected; OR = 10.79, SE = 0.392, *p* < 0.0001) and the lowest detection probability was in striped crayfish snakes (0/10 with *Hepatozoon* spp. detected; OR = 0.1098, SE = 0.810, *p* = 0.0064; *Liodytes alleni*) and Florida green watersnakes (4/35 with *Hepatozoon* spp. detected; OR = 0.1789, SE = 0.440, *p* < 0.0001; Table 5 and Figure 3). *Mycoplasma* spp. detection probability was highest in ring-necked snakes (12/35 with *Mycoplasma* spp. detected; OR = 0.4361, SE = 0.331, *p* = 0.0121) and lowest in black swamp snakes (0/15 with *Mycoplasma* spp. detected; OR = 0.0892, SE = 0.533, *p* < 0.0001; Table 5 and Figure 3). *Ro* detection probability was highest in pygmy rattlesnakes (14/34 with *Ro* detected; OR = 0.5885, SE = 0.307, *p* = 0.0844) and lowest in Florida green watersnakes (0/32 with *Ro* detected; OR = 0.0218, SE = 0.862, *p* < 0.0001) and ring-necked snakes (0/20 with *Ro* detected; OR = 0.0305, SE = 0.932, *p* = 0.0002; Table 6 and Figure 3). Pooled age class was further associated with pathogen detection, with detection consistently lower in juveniles than in adults (Table 4).

Strong spatial trends were observed with the detection of *Oo, Mycoplasma* spp., *Cryptosporidium* spp., and *Ro*. For *Oo*, the predicted detection probability was highest at the opportunistic Georgia site (OR = 7.242, SE = 0.523, *p* = 0.0002) compared with the Florida site (Table 4). For *Hepatozoon* spp., the predicted detection probability was lowest at the South Carolina site (OR = 0.4743, SE = 0.292, *p* = 0.0105) compared with the Florida site (Table 4). *Mycoplasma* spp. were almost exclusively detected at the Florida site (28.2%, 95% CI: 23.1–34.1), with only one detection at the South Carolina site (0.8%, 95% CI: 0.1–4.2), and the remaining detections from Athens-Clarke County, Georgia (*n* = 2), Barnwell County, South Carolina (*n* = 1), Hardin County, Tennessee (*n* = 1), and Miami-Dade County, Florida (*n* = 1; Table 4). Additionally, most *Cryptosporidium* spp. detections were in Florida (*n* = 8) with two detections at the South Carolina site (Table 4). *Ro* detections were only in snakes from Florida (Table 4). Site was not significantly associated with the detection of *S. enterica*. The prevalence of snakes with no pathogen detections across sites was similar [Florida: 19.4% (56/288), South Carolin: 18.8% (27/144), Georgia opportunistic: 16.7% (5/30)].

**Figure 4:**
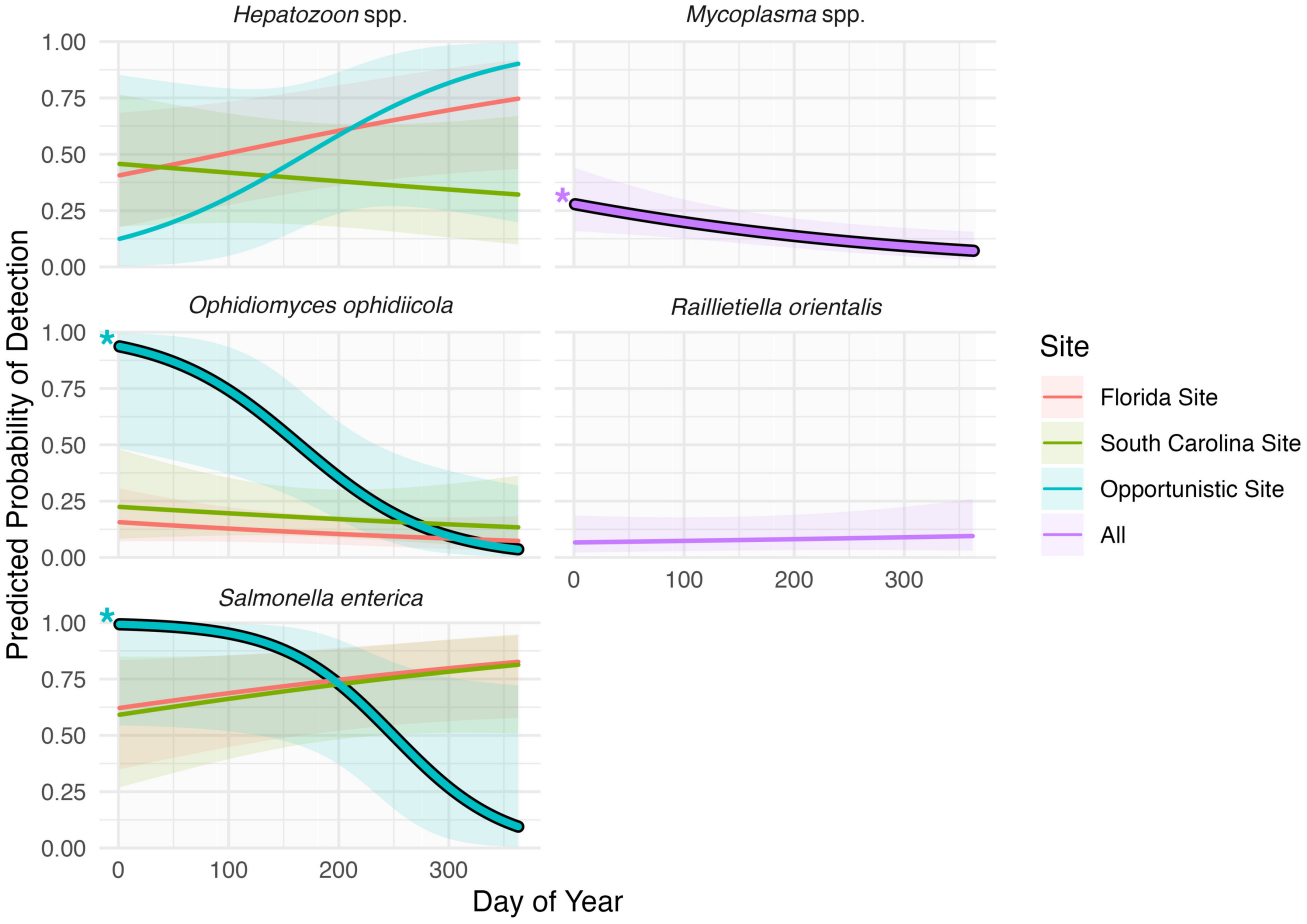
Spatiotemporally predicted pathogen detection probability using generalized linear mixed models in free-ranging snakes sampled from the southeastern United States from 2021 to 2024. Bold curves denoted by asterisks (*) indicate significant trends (*p* ≤ 0.05). Day 1 is defined as the first day of winter (December 21). *Raillietiella orientalis* analysis was limited to snakes from Florida.

Moderate temporal trends of *Mycoplasma* spp. predicted the highest detection rates in winter, with rates gradually decreasing through spring and summer into fall (OR = 0.6894, SE = 0.139, *p* = 0.0075; Figure 4). Detection of *Oo* exhibited a site-specific temporal trend at our opportunistic Georgia site, with *Oo* detection highest in winter, with rates gradually decreasing through spring and summer into fall (OR = 0.3056, SE = 0.598, *p* = 0.0475; Figure 4). *Salmonella enterica* detection had a site-specific temporal trend at our opportunistic Georgia site with *S. enterica* detection highest in winter, with rates decreasing through spring and summer into fall (OR = 0.1475, SE = 0.897, *p* = 0.0295, Figure 4). DOY was not significantly predictive of *Hepatozoon* spp. (OR = 1.402, SE = 0.182, *p* = 0.0634) or *Ro* (OR = 1.094, SE = 0.160, *p* = 0.5750) detection (Figure 4). Recapture status was only predictive of *Hepatozoon* spp. detection (OR = 3.286, SE = 0.437, *p* = 0.0065), with most also having *Hepatozoon* spp. detected at recapture. Recapture status is further summarized in Supplementary Figure 4.

### 3.5 Physical exam analysis

**Figure 5:**
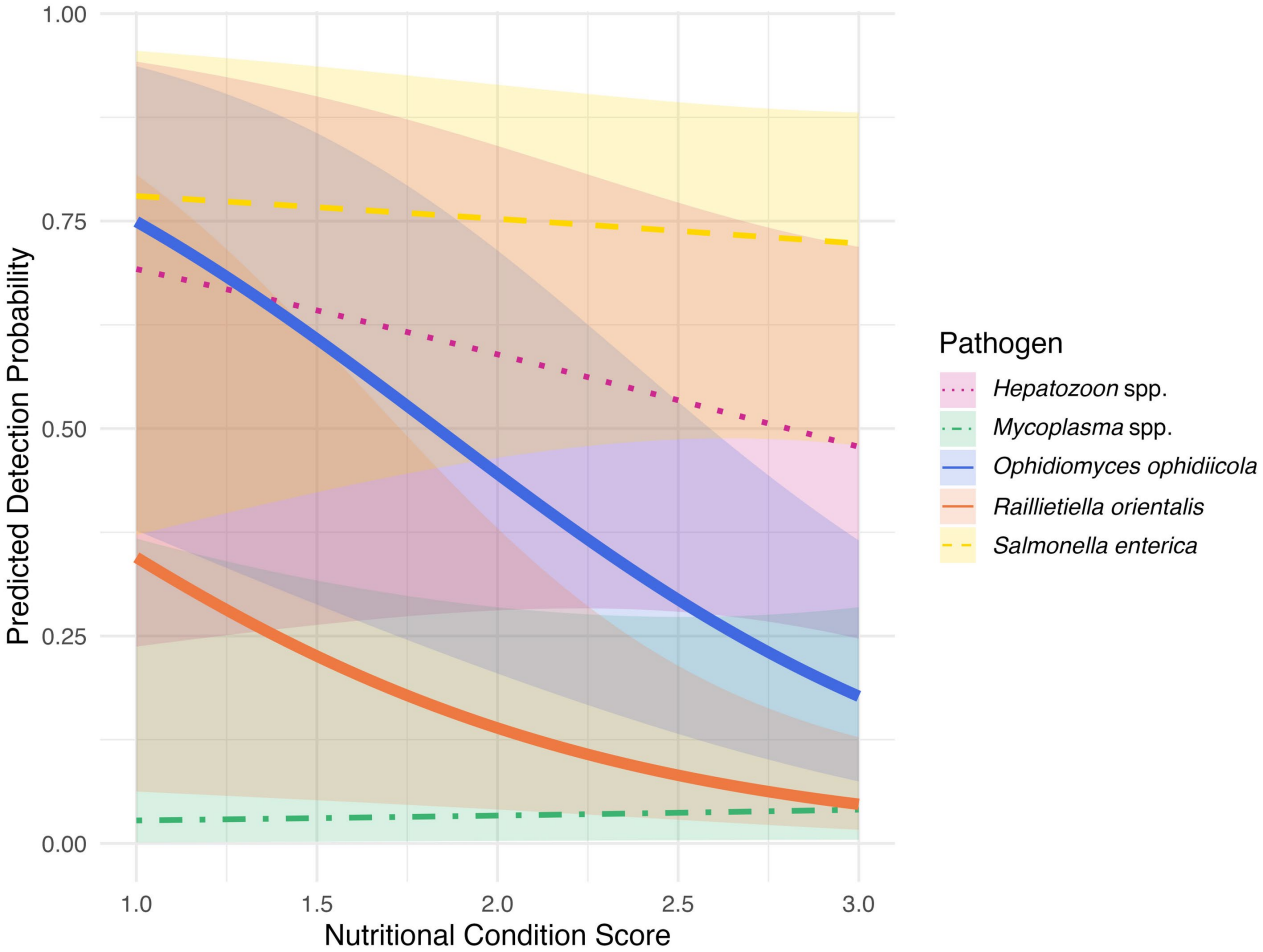
Predicted probability of pathogen detection in relation to nutritional condition score using generalized linear mixed models in free-ranging snakes from the southeastern United States sampled from 2021 to 2024. Thicker lines indicate statistically significant (*p* < 0.05) predictors (*Ophidiomyces ophidiicola* and *Raillietiella orientalis*). *Raillietiella orientalis* analysis was limited to snakes from Florida.

Most snakes examined, 90.8% (462/509), were in good nutritional condition with a score of 3. However, numerous snakes exhibited lower scores, including 11 with a score of 2.5; 31 with a score of 2; one with a score of 1.5; and four with a score of 1 (indicating extreme emaciation). Nutritional condition score was negatively associated with *Oo* (OR = 0.2685, SE = 0.351, *p* = 0.0002) and *Ro* detection (OR = 0.3070, SE = 0.508, *p* = 0.0200; Figure 5). Furthermore, snakes with higher nutritional condition scores (score = 3) had ~70% decreased odds of *Ro* and/or *Oo* detection *versus* those with a score of <3. Nutritional condition score was not significantly associated with detection of *Hepatozoon* spp., *Mycoplasma* spp., or *S. enterica.* All snakes in which *Cryptosporidium* spp. was detected received good nutritional condition scores. Clinical signs consistent with respiratory disease (e.g., open-mouth breathing and rostral/naris crusting) were documented in 1.6% of snakes examined (8/505, 95% CI: 0.8–3.1%) and were strongly associated with *Oo* detection (OR = 36.53, SE = 1.223, *p* = 0.0033). No other pathogen detections were associated with respiratory clinical signs. Pathogen detection was not significantly associated with sex (Table 4).

### 3.6 Skin lesion severity analysis

Skin lesions (e.g., misshapen scales, crusting, ulceration) were observed in 45.9% (233/508, 95% CI: 41.6–50.2%) of snakes. Among those with such lesions, *Oo* was detected in 30.5% (71/233, 95% CI: 24.9–36.7%). Conversely, *Oo* was detected in 2.0% (10/508, 95% CI: 1.1–3.6%) of snakes without skin lesions, representing 12.2% (10/82, 95% CI: 6.8–21%) of *Oo* detections. Mean skin lesion severity score was 6.9, with scores ranging from 0 to 12, which represents the full range of possible scores. Full-body skin swab *Oo* DNA extract concentration (*Oo* DNA copies per ng DNA extract) was positively associated with skin lesion severity score (RR = 1.039, SE = 0.014, *p* = 0.0069; Supplementary Figure 5). Species was predictive of skin lesion severity score, with black racers (RR = 7.875, SE = 0.085, *p* < 0.0001) having the highest predicted scores and banded watersnakes (RR = 5.631, SE = 0.082, *p* < 0.0001) having the lowest. Lesion severity was not significantly associated with coinfection presence, DOY, site, or recapture status.

### 3.7 Ophidiomycosis case classification analysis

Most snakes at initial capture were classified as “negative” (51.9%, 264/509, 95% CI: 47.5–56.2; *Oo* not detected and no skin lesions), with 31.8% snakes classified as “possible” (162/509, 95% CI: 27.9–36.0; skin lesions present but *Oo* not detected), 13.9% as “apparent” (71/509, CI: 11.2–17.2; skin lesions present and *Oo* detected), 2.0% as “present” (10/509, 95% CI: 1.1–3.6; no skin lesions but *Oo* detected) with the remaining two snakes not classified due to poor carcass condition. Snakes in the “present” category were excluded from the analysis due to low sample size. Case classification MLRM analysis revealed significant trends in ophidiomycosis case classification temporally (DOY), spatially (site), by coinfection status, and by species.

Apparent and possible ophidiomycosis were most likely detected in winter, with probabilities decreasing progressively through spring, summer, and fall (apparent: OR = 0.6161, SE = 0.160, *p* = 0.0025, possible: OR = 0.6864, SE = 0.118, *p* = 0.0014). Spatially, apparent ophidiomycosis had higher predicted odds at the South Carolina site (OR = 2.653, SE = 0.452, *p* = 0.0307) and the opportunistic Georgia site (OR = 10.62, SE = 0.670, *p* = 0.0004) than at the Florida site. Coinfected snakes were ~11 times more likely to be classified with apparent ophidiomycosis (OR = 11.19, SE = 0.387, *p* < 0.0001) and ~2 times more likely to be classified as possible ophidiomycosis (OR = 2.059, SE = 0.248, *p* < 0.0036) than snakes classified as “negative.”

Pygmy rattlesnakes were more likely to have apparent ophidiomycosis than black racers (OR = 1.427, SE = 0.090, *p* = 0.0148), banded watersnakes (OR = 1.354, SE = 0.0852, *p* = 0.0396), and eastern ribbon snakes (OR = 1.535, SE = 0.0983, *p* = 0.0051). Black racers were more likely to have possible ophidiomycosis than ring-necked snakes (OR = 1.594, SE = 0.094, *p* = 0.0010), black swamp snakes (OR = 1.582, SE = 0.120, *p* = 0.0211), banded watersnakes (OR = 1.549, SE = 0.072, *p* < 0.0001), Florida green watersnakes (OR = 1.552, SE = 0.095, *p* = 0.0024), eastern rat snakes (OR = 1.595, SE = 0.106, *p* = 0.0046), pygmy rattlesnakes (OR = 1.622, SE = 0.083, *p* < 0.0001), and common garter snakes (OR = 1.603, SE = 0.101, *p* = 0.0021). Eastern ribbon snakes were more likely to have possible ophidiomycosis than banded watersnakes (OR = 1.385, SE = 0.087, *p* = 0.0239) and pygmy rattlesnakes (OR = 1.450, SE = 0.102, *p* = 0.0318). Ring-necked snakes (OR = 1.481, SE = 0.104, *p* = 0.0224) and banded watersnakes (OR = 1.470, SE = 0.0746, *p* = 0.0006) were more likely to be negative for ophidiomycosis than black racers.

### 3.8 Mortality events and postmortem examinations

**Figure 6:**
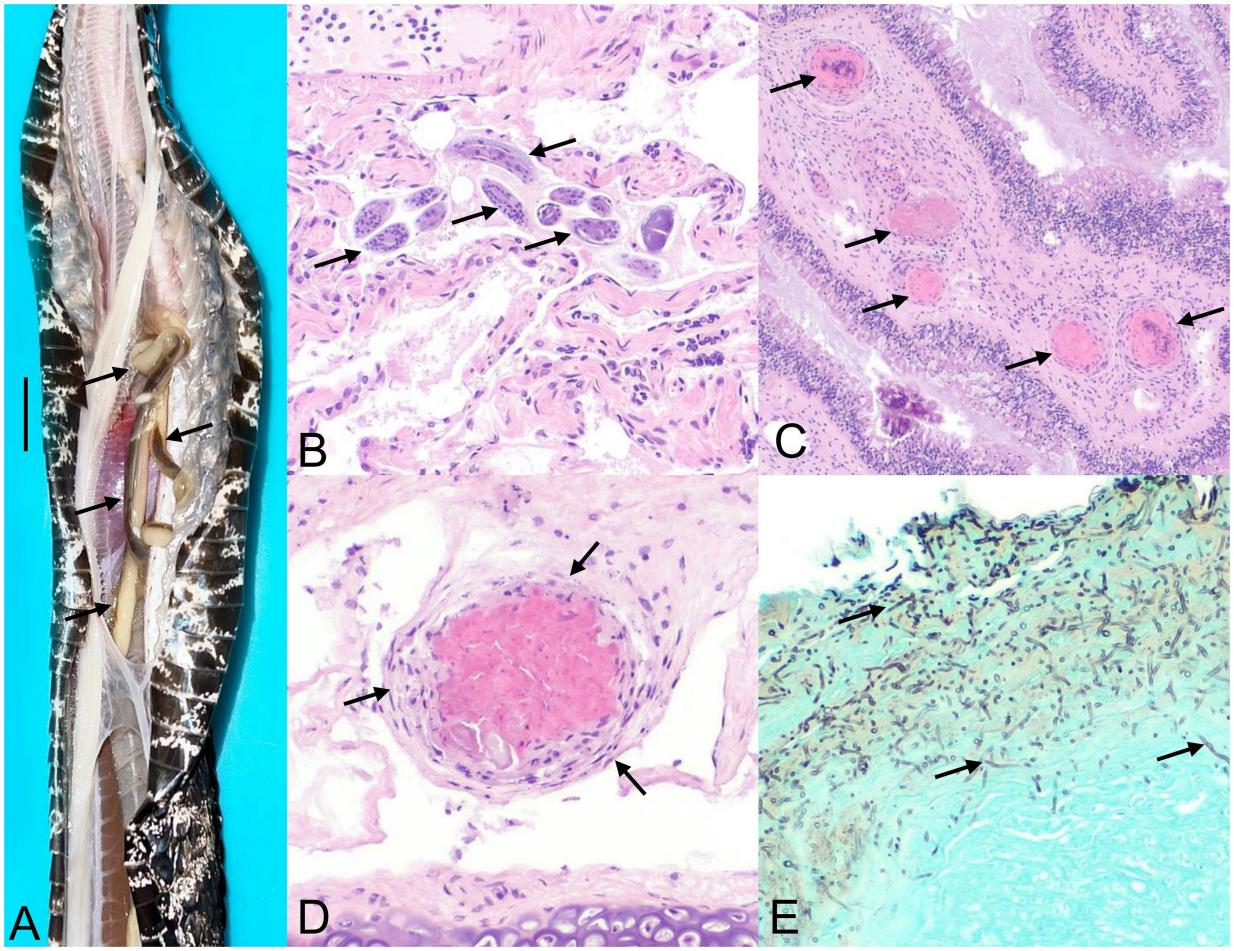
Gross and histological findings in opportunistically evaluated carcasses. (**A**; left, scale bar = 1 cm) adult *Raillietiella orientalis* (arrows) within the lungs and coelomic cavity of an adult pygmy rattlesnake (*Sistrurus miliarius*). (**B**; top middle) photomicrograph (10×; hematoxylin and eosin stain) of lung from an adult common garter snake (*Thamnophis sirtalis*) with numerous cross and longitudinal sections of larval nematodes (arrows) in a faveolus surrounded by edema. (**C**; top right) photomicrograph (4×; hematoxylin and eosin stain) of intestine from an adult pygmy rattlesnake with a focal heterophilic granuloma (arrows) in a septum. (**D**; bottom middle) photomicrograph (10×; hematoxylin and eosin stain) of trachea from an adult pygmy rattlesnake with multifocal subepithelial granulomas (arrows) of suspected parasitic origin (based on central contents in some granulomas, not depicted). (**E**; bottom right) photomicrograph (10×; GMS stain) of head skin from an adult pygmy rattlesnake showing numerous fungal silver-stained (gray-black) hyphae (arrows) within the superficial to mid dermis, with overlying epithelial ulceration.

Final diagnoses from postmortem cases (*n* = 61; 53 full examinations, 8 partial examinations) are summarized in Table 7. Overall, the most common cause of death for snakes examined postmortem was trauma, specifically from suspected vehicular collision (41%, 25/61). From November 2022 to March 2023 at the Florida refuge site, one common garter snake and eight pygmy rattlesnakes were found dead or moribund with no external signs of trauma. Among these, one common garter snake and five pygmy rattlesnakes were suitable for postmortem examination, which revealed poor nutritional condition (minimal to no adipose and atrophied musculature) in 5/6 (83%) snakes. All six snakes had pentastomiasis with 4–12 (mean = 8.7) adult *Ro* in their oral cavity, trachea, lungs, and/or free in the coelomic cavity (Figure 6). Three snakes had confirmed ophidiomycosis (*Oo* detected concurrent with skin lesions with fungal hyphae on histopathology), and a fourth had evidence of non-*Oo* dermatomycosis (*Oo* not detected via PCR, but fungal hyphae histologically evident; Figure 6). *Salmonella enterica* and *Hepatozoon* spp. were detected in all six snakes, one of which had clinical hepatozoonosis based on severe and disseminated histologic lesions associated with developmental stages of the parasite (Figure 6). One snake had *Mycoplasma* spp. detected. All snakes had disseminated parasite infestations, including unidentified nematodes (*n* = 3), trematodes (*n* = 2), and cestodes (*n* = 1, Figure 6). Two snakes also had pneumonia, one being verminous and the other bacterial (Figure 6). Three snakes were recaptures, with initial capture from 19 to 33 days before being found dead or moribund. Body mass sharply declined during this period (i.e., 16.0–21.6% loss). Collectively, postmortem evaluation revealed systemic disease in all snakes, associated with the hematophagous parasite *Ro*.

**Table 7:** Diagnoses (i.e., causes and contributors to death) for snakes collected from across the southeastern United States from 2021 to 2024 that underwent postmortem evaluation.

Final diagnosis	*n*
Trauma cases	
Blunt force trauma (suspected vehicular collision)	21
Blunt force trauma (suspected vehicular collision), ophidiomycosis	2
Blunt force trauma (suspected vehicular collision), pentastomiasis	1
Blunt force trauma (suspected vehicular collision), dermatomycosis, gastrointestinal parasitism, poor nutritional condition, trematodiasis	1
Suspected trauma (ant predation)	1
Trauma (suspected predation)	4
Trauma (suspected predation), gastrointestinal parasitism	1
Trauma (suspected predation), nematodiasis	1
Trauma (entrapment)	2
Trauma (entrapment), gastrointestinal parasitism	1
Trauma (entrapment), ophidiomycosis	1
Penetrating trauma (entrapment), gastrointestinal parasitism, pentastomiasis	1
Ophidiomycosis cases	
Ophidiomycosis	2
Ophidiomycosis, bacterial septicemia, gastrointestinal parasitism	1
Ophidiomycosis, gastrointestinal parasitism, poor nutritional condition	1
Pentastomiasis cases	
Pentastomiasis, disseminated (multisystemic) parasitic infection, dermatomycosis	1
Pentastomiasis, poor nutritional condition, verminous pneumonia	1
Pentastomiasis, disseminated (multisystemic) parasitic infection	1
Pentastomiasis, ferlaviral disease, dermatomycosis, nematodiasis, poor nutritional condition	1
Ophidiomycosis, pentastomiasis comorbidities	
Ophidiomycosis, pentastomiasis, bacterial pneumonia, disseminated (multisystemic) parasitic infection, poor nutritional condition	1
Ophidiomycosis, pentastomiasis, disseminated (multisystemic) parasitic infection, poor nutritional condition	1
Ophidiomycosis, pentastomiasis, disseminated (multisystemic) parasitic infection, hepatozoonosis, poor nutritional condition	1
Other cases	
Enteritis (suspected viral origin) with secondary bacterial infection, gastrointestinal parasitism	3
Hepatozoonosis, emaciation (starvation)	2
Disseminated (multisystemic) disease (suspect viral)	1
Enteritis (suspected viral origin) with secondary bacterial infection	1
Suspected gastrointestinal tract disease	1
Dermatomycosis, poor nutritional condition	1
Poor nutritional condition	1
*Cryptosporidium serpentis* and *Ophidiomyces ophidiicola* detected	1
No evidence of disease	2
Total	61

The remaining 55 snakes evaluated postmortem included 11 snakes from the Florida site, 11 from the South Carolina site, and 33 submitted to SCWDS. The cause of death for snakes from the Florida site was most often attributed to trauma [vehicular collision (*n* = 1), predation (*n* = 1), or trap-associated (*n* = 5)]. Among snakes that died from trauma, concurrent diagnoses included ophidiomycosis (*n* = 1), pentastomiasis (*n* = 1, coinfection with *Ro* and *Kiricephalus coarctatus*), and gastrointestinal parasitism (*n* = 2). Diagnoses in snakes found dead or moribund without signs of trauma included poor nutritional condition (*n* = 2), dermatomycosis (*n* = 1), ophidiomycosis (*n* = 1), and suspected gastrointestinal tract disease (*n* = 2). One snake diagnosed with ophidiomycosis, poor nutritional condition, and gastrointestinal parasitism was a recapture with a 26.9% decline in body mass from the first capture. Mortality of the 11 snakes from the South Carolina site was attributed to trauma with nine attributed to presumed vehicular collision (i.e., found dead on the road) and two attributed to presumed predation (one found decapitated and the other in an ant hill). Two of these snakes also had gastrointestinal parasitism, one of which also had dermatomycosis, poor nutritional condition, and trematodiasis.

Of the 33 snakes submitted to SCWDS, cause of death was attributed to infectious disease(s) in 13 snakes, and trauma in 20 snakes, usually via blunt force (*n* = 15; suspected vehicular collision; found dead on the road) or predation (*n* = 4). Diagnoses among snakes without trauma included enteritis (*n* = 4; suspected viral origin with secondary bacterial infection), gastrointestinal parasitism (*n* = 4), hepatozoonosis (*n* = 2), ophidiomycosis (confirmed: *n* = 2; *Oo* detected: *n* = 1; apparent: *n* = 1), poor nutritional condition (*n* = 4), *C. serpentis* detected (*n* = 1), disseminated disease (*n* = 1; suspected viral), bacterial septicemia (*n* = 1), ferlaviral disease (*n* = 1), nematodiasis (*n* = 1), and pentastomiasis (*Ro*, *n* = 1). Additional diagnoses among snakes with trauma included confirmed ophidiomycosis (*n* = 2), nematodiasis (*n* = 1), and pentastomiasis (*Ro*, *n* = 1).

## 4 Discussion

We conducted surveillance for seven pathogens in free-ranging snakes and performed risk factor analyses that revealed important epidemiological trends. Our results demonstrate that apparent ophidiomycosis (i.e., characteristic lesions and *Oo* detection via qPCR) was strongly associated with coinfections in free-ranging snakes from the southeastern United States. Detection of *Oo* and *Ro* was further associated with poor nutritional condition, suggesting negative impacts on snake health. This study also provides insight into the epidemiology of several understudied pathogens in these snakes. For example, *Mycoplasma* spp. were detected in nearly 20% of sampled snakes, which, to our knowledge, has not been reported previously in free-ranging snakes (57). We found high detection prevalence of the endemic pathogens *Hepatozoon* spp. [53.4% (205/384)] and *S. enterica* [62.6% (306/489)] (90, 91). *Cryptosporidium* spp. were rarely detected, consistent with recent surveillance in multiple free-ranging snake species throughout the southeastern United States (66). Our data enhance understanding of two significant health threats in snake populations, the invasive pentastome *Ro* and the globally distributed fungal pathogen *Oo*, and set a baseline for other pathogens of interest (6, 58, 59, 92).

Of the snake species examined in our study, pygmy rattlesnakes, sampled exclusively from the Florida site, exhibited consistently high pathogen detection prevalences (*Oo*, *Ro*, and *S. enterica*) and apparent ophidiomycosis. Inclusion of opportunistic postmortem data enhanced our assessment of health concerns in this species. Collectively, our findings suggest high susceptibility to coinfections and potential disease-related population impacts in pygmy rattlesnakes. This is further supported by historical reports of mycosis outbreaks in the 1990s (likely ophidiomycosis before its recognition) and more recent documentation of *Oo*- and *Ro-*related mortalities at the same Florida site (24, 39, 60, 61, 93). Earlier studies did not include other snake species and therefore could not assess species-specific pathogen or disease patterns (61, 93). Our findings contribute to mounting evidence for population-level concerns based on infectious and parasitic diseases in pygmy rattlesnakes, specifically at our Florida site, potentially influenced by environmental stressors including habitat destruction, fragmentation, and climate change. These findings underscore the need for targeted conservation and monitoring of pygmy rattlesnakes to inform population trend assessments and mitigate further declines.

Among all surveyed species, the banded watersnake was the most frequently captured species at both sites and consistently exhibited low *Oo* and *Ro* detection prevalences, lower skin lesion severity scores, and a lower likelihood of apparent ophidiomycosis compared with other species such as pygmy rattlesnakes and black racers. These findings highlight important species-specific differences that may reflect regional population dynamics. Previous studies on federally protected snakes, including the eastern indigo snake and eastern massasauga rattlesnake (closely related to pygmy rattlesnakes), have reported increased risk of apparent ophidiomycosis and higher likelihood of *Oo-*associated mortality (7, 18, 94). Similarly, in Texas, the threatened Brazos watersnake (*Neroida harteri harteri*) shows higher *Oo* detection prevalence than the sympatric, more stable diamondback watersnake (*Nerodia rhombifer*) (30). These observations suggest that snake populations already considered of conservation concern due to factors such as low genetic diversity, specialized habitat requirements, or increased human persecution may be at heightened risk of pathogen exposure and disease development (8, 9, 18, 94).

General antemortem health assessments, combined with opportunistic postmortem evaluations, suggest that *Oo* and *Ro* represent important health threats to southeastern US snake populations. Supporting findings include poor nutritional condition concurrent with *Oo* and/or *Ro*, in addition to the documented mortality event at the Florida site discussed above. The recent invasion of *Ro* into native snake populations in Florida represents a novel threat that may synergize with the more established pathogen *Oo* and merits increased conservation concern and continued monitoring for infection and disease in native snakes in the region (59, 61, 95). Our finding of emaciation in snakes with ophidiomycosis is consistent with previous findings and suggests that ophidiomycosis may impair snakes’ ability to forage, thermoregulate, and perform other essential functions (6, 14, 39, 96). Furthermore, negative energy balance likely affects snake immune function and the ability to resist both more severe ophidiomycosis and additional pathogen invasion. Similarly, *Ro* infection may contribute to emaciation, particularly with high burdens in the respiratory tract. Because *Ro* is hematophagous, it may contribute to anemia, further weakening the host (97). Both *Oo* and *Ro* can cause chronic infections in snakes; thus, it is possible that *Oo* causes persistent or repeated infections due to environmental exposures and/or incomplete clearance through ecdysis, whereas *Ro* may be difficult for the host to eliminate, resulting in repeated infections and cumulative parasite burdens (6, 59, 98). Improved understanding of the long-term population health effects of these infections, particularly *Ro* and *Oo* coinfections, is needed. This is especially critical following the introduction of *Ro* to South Florida, given its demonstrated ability to spread beyond its introduced range (61, 95).

The number of snakes with skin lesions was higher than that from which *Oo* was detected in our study, which is consistent with previous *Oo* surveillance studies (18, 70, 99). Lack of *Oo* detection in swab or lesion samples may reflect alternative causes of lesions, such as bacterial or non-*Oo* fungal infections or traumatic injury, or may indicate that fungal infection has extended into deeper tissues or been cleared from the skin (13, 18, 70). Recent detections of a related fungus, *Paranannizziopsis* spp., in the southeastern United States raise concerns regarding alternative fungal etiologies for dermatomycosis in free-ranging snakes, necessitating broader surveillance and diagnostic evaluation (100, 101). Investigations of other fungal etiologic agents, such as *Paranannizziopsis* spp., are also warranted, as many snakes (e.g., black racers) frequently exhibited lesions consistent with dermatomycosis without concurrent *Oo* detection.

Evaluation of the common endemic pathogens *Hepatozoon* spp. and *S. enterica* revealed high detection prevalence for both. The high detection prevalence of *S. enterica* in free-ranging snakes throughout the Southeast suggests that it may be part of the normal gastrointestinal flora in these snakes and, as a potential zoonotic pathogen, highlights the importance of biosafety practices when handling reptiles (90). *Hepatozoon* spp. detection prevalence was consistent with previous reports in the southeastern United States (53, 54). Additionally, host taxonomic trends in *Hepatozoon* spp. detection were similar to those previously reported, with banded watersnakes having higher predicted detection probabilities than Florida green watersnakes (53, 54). Differences in detection may be related to diet, as snakes with lizard- and anuran-based diets appear to have higher prevalence than species with piscivorous diets (102). Clinical manifestations may also reflect parasite burden and/or comorbidities (103, 104).

Similar to *Salmonella* spp. detections, *Mycoplasma* spp. detections in free-ranging southeastern snakes were apparently subclinical, with an overall detection prevalence of 17.5% (78/445) and no associations with nutritional condition scores or clinical respiratory signs. This supports the currently accepted view of *Mycoplasma* spp. as a common commensal bacterium in snakes (57, 105). Consistent with prior studies in the region, *Cryptosporidium* spp., primarily *C. serpentis*, at low prevalence and appeared subclinical (66). However, *Cryptosporidium* spp. have been detected at higher prevalences (≤33.3%) in free-ranging snakes in Japan and 21.0% (*n* = 28) in eastern indigo snakes in southern Florida (65, 106, 107). Serpentoviruses were not detected in any sampled individuals, suggesting that populations sampled at our refuge sites may be naïve or that detection was limited by challenges associated with field-based RNA virus surveillance, including RNA degradation and the highly divergent nature of serpentoviruses (63).

Apparent ophidiomycosis was strongly associated with coinfections across multiple snake species. These findings, together with postmortem results, suggest that coinfections may increase susceptibility to *Oo* infection and disease development. Conversely, the presence of ophidiomycosis may increase susceptibility to secondary infections. Although causality cannot be determined from this study, previous studies highlight the importance of coinfections in snakes and their potential role in facilitating infections such as *Cryptosporidium* spp. (108), serpentoviruses, and *Mycoplasma* spp. infections and associated disease development (105). Although parasites are ubiquitous in ecosystems and often subclinical in wildlife vertebrate hosts, the introduction of novel pathogens into systems can be detrimental to hosts due to lack of coevolution (109). Environmental stressors, including climate change and anthropogenic disturbances, may further exacerbate disease outcomes.

Spatiotemporal analyses revealed several important patterns, including detection of *Ro*, *Cryptosporidium* spp., and *Mycoplasma* spp., primarily in Florida. The restricted detection of *Ro* to Florida snakes aligns with its currently recognized US distribution (95), although its rapid spread raises concern for further expansion. Geographic distributions of *Mycoplasma* spp. in free-ranging US snakes have not been previously investigated; however, increased detection in Florida raises concerns about whether these pathogens are endemic or introduced via potential spillover from the numerous invasive species in Florida (110). Snakes in our opportunistic Georgia site had a significantly higher *Oo* detection prevalence compared with Florida sites, potentially due to lower seasonal temperatures in the former sites being more conducive to fungal growth (4). Our results further support documented region-specific, seasonal *Oo* epidemiologic patterns in the United States, with detection risk peaking in winter and gradually declining throughout the remainder of the year (24, 39, 67, 94, 111). Unlike most existing studies on snake pathogens, we analyzed seasonal trends for *S. enterica*, *Hepatozoon* spp., *Ro*, and *Mycoplasma* spp. detection. *Mycoplasma* spp. exhibited a significant seasonal pattern with greater detection prevalence in winter. *Salmonella enterica* displayed a significant site-specific seasonal trend at our opportunistic Georgia site (with higher detection in winter). *Hepatozoon* spp. showed a non-significant tendency toward higher detection prevalence in summer and fall. The detection prevalence of *Ro* did not exhibit a clear seasonal trend. Further investigation into environmental and host drivers of pathogen transmission and disease development is needed to evaluate these trends. For example, transmission of ingested pathogens, such as *Hepatozoon*, may have seasonal influences based on foraging behavior, prey preferences, and food availability, which may be influenced by seasonal temperatures, humidity, and host responses to these conditions (90, 102). Recapture data from our study support persistent detection of *Hepatozoon* spp., as snakes with *Hepatozoon* spp. detected at initial capture were more likely to have *Hepatozoon* spp. detected at subsequent samplings. In contrast, detection status for the other pathogens included in this study varied within individuals over time.

Non-invasive wildlife surveillance is challenging but worthwhile for establishing baseline prevalences in understudied populations and guiding future research and diagnostic efforts. For example, our results suggest that paired fecal and cloacal swab samples are likely most effective for detecting multiple enteric pathogens and parasites, given the poor agreement between sample types for detection of selected gastrointestinal pathogens. Moreover, we observed increased detections of *S. enterica* via cloacal swabs; however, feces had higher detection of *Ro* and *Cryptosporidium* spp. compared with cloacal swabs. Superior *Ro* detection via PCR testing of feces *versus* cloacal swabs has been reported, with a test sensitivity of 97.7% for feces and 21.7% for cloacal swabs (73). Given these findings, *Ro* prevalence is likely most accurately determined by fecal wet mount with concurrent molecular identification. Studies investigating sample sensitivity for *C. serpentis* detection have shown that gastric swabs, gastric washes, or gastric biopsies are the most sensitive (100% sensitivity), with reduced sensitivity (72%) for cloacal swabs (112, 113). Based on previous studies, the reported prevalences for *Cryptosporidium* spp. and *Ro* in our study are likely underestimated due to sampling limitations in live snakes, as well as limitations in *Ro* detection via cloacal swabs, because feces were not always available (114). Increased detection of *S. enterica* in cloacal swabs compared with feces is supported by previous studies in Burmese pythons (115).

There are numerous limitations in sampling and assessing the health of free-ranging snakes. These include potential biases from different trapping or capture techniques, with the most efficient methods often tailored to specific field sites and study goals. Additionally, field methods are often biased toward certain snake species, with additional challenges in collecting predominantly aquatic snakes. Our study was primarily conducted in two wetland sites and thus best represents species that are abundant in those habitats. Studies focused on other important snake habitat types, such as the longleaf pine ecosystem, are needed to better represent additional species. Furthermore, collecting a complete set of biological samples is challenging for smaller individuals and species (e.g., limited blood collection volume) as well as venomous species (e.g., restricted sampling, such as choanal swabs, and additional safety precautions). Some samples, such as feces, are not available from every sampled snake (due to extended periods between meals), which limited opportunities for *Ro* detection in our study with increased detections likely in postmortem cases. Discrepancies in pathogen-specific and coinfection sample sizes in our dataset reflect incomplete sample sets for some snakes, due to limitations related to body size, safety, and opportunistic sampling. Antemortem nutritional condition assessment is further limited by inherent subjectivity and the inability to directly quantify coelomic adipose stores and musculature.

Future directions in snake health studies highlighted by our results include the taxonomic classification and investigation of risk factors associated with *Hepatozoon* and *Mycoplasma* spp. infections. There likely are additional species within these genera that remain unidentified due to limited available sequence data. Further pathogen classification may prove useful when considering host species selection, disease outcomes, and spatial trends. Additional investigations into coinfections are warranted, including, when possible, postmortem examination for more comprehensive disease assessment. Coinfections involving multiple pathogen combinations (e.g., helminth parasites, viruses, fungi) were diagnosed postmortem in our study and may have synergistically contributed to poor snake health and systemic disease. Additional population health investigations evaluating associations between detection of *Oo, Ro,* and physical examination findings would further elucidate how at-risk populations may be affected by mortality outbreaks.

Snakes face the added threat of human persecution in addition to threats shared by many wildlife species, such as climate change and habitat destruction (116–118). Our study highlights additional conservation challenges, such as the introduction of invasive parasites, and provides novel insights into the epidemiology of important pathogens of free-ranging snakes, including concurrent infections, in the southeastern United States. Our methods can be applied to future snake studies targeting additional species and regions, and our data can inform conservation management programs. The latter may include translocation, quarantine, and reintroduction of snakes, as well as the development of biosafety protocols. Our holistic approach to snake health provides a model for future research to inform conservation management strategies for snakes and other wildlife species.

## Data Availability

The datasets presented in this study can be found in online repositories. The names of the repository/repositories and accession number(s) can be found in the article/Supplementary material. The datasets generated and analyzed for this study can be found in the University of Georgia’s Open Scholar research data repository (https://openscholar.uga.edu/record/27961?&ln=en), DOI: https://doi.org/10.71927/uga.27961.
