# Supplementary material for: Health assessment and multipathogen surveillance of free-ranging snakes in the southeastern United States
Source: Front Vet Sci. 2026 May 26;13:1754420. doi: 10.3389/fvets.2026.1754420 (PMC13248790; doi:10.3389/fvets.2026.1754420)
Supplement: Supplementary file 1 [file Table_1.docx]

Supplementary Material

# Supplementary Phylogenetic Analysis Methods

Phylogenetic analyses for *Hepatozoon* and *Mycoplasma* spp. sequences were performed in Geneious Prime^®^ 2025.1.1 (1). MUSCLE 5.1 (2) was used for multiple alignment for both maximum likelihood estimation (RAxML (3); GTR GAMMA as the nucleotide model, default settings and a bootstrap analysis of 1000 repeats) and Bayesian phylogenetic analysis (MrBayes v3.2.6 (4); default settings). Samples were clustered according to consensus identity, those that were either identical or had <10 nucleotide difference were grouped with a representative consensus sequence used for phylogenetic analysis. Reference sequences were selected from GenBank^®^ based on the closest nucleotide BLAST^®^ matches (5).

# Supplementary Statistical Analysis – Carcass versus Live Snake Samples

We analyzed pathogen detection in association with ‘Sampling Type’ (Live vs. Carcass) both using a univariable binomial generalized linear model (function glm) and as a covariate in the primary multivariable binomial generalized linear mixed-effects model (R package lme4, function glmer (6)). In this analysis, ‘Sampling Type’ was not associated with detection of *Ophidiomyces ophidiicola* (univariable model *P*=0.257, multivariable model *P*=0.244), *Salmonella enterica* (univariable model *P*=0.802, multivariable model *P*=0.774), *Hepatozoon* spp. (univariable model *P*=0.0598, multivariable model *P*=0.932), or *Mycoplasma* spp. (univariable model *P*=0.279, multivariable model *P*=0.968). However, ‘Sampling Type’ was associated with *Raillietiella orientalis* (*Ro*) detection (univariable model *P*=0.0413, multivariable model *P*=0.000649). A potential explanation for the significant association between *Ro* detection and ‘Sampling Type’ is that there was an ongoing mortality event associated with *Ro* at our Florida field site during sampling (as described in our manuscript). Thus, we pooled sample type for the analyses performed in the primary manuscript.

# Supplementary Figures

## Supplementary Figures Legends

**Supplementary Figure 1.** *Hepatozoon* phylogenetic tree based on 18S rRNA sequences (600 bp). Sequences from *Hepatozoon* spp. PCR detections in free-ranging snakes from the southeastern United States are grouped in clusters (tip labels in bold, representing current study samples) followed by the number of sequences represented by each excluding recaptures. The topology tree was inferred by Bayesian and maximum-likelihood methods with the maximum-likelihood represented. *Adelina grylli* (DQ096836.2) was used as the outgroup (7). Icons at right indicate the host taxa from which the *Hepatozoon* spp. was detected.

**Supplementary Figure 2.** *Mycoplasma* phylogenetic tree based on IGS sequences (722 bp). Sequences from *Mycoplasma* spp. PCR detections in free-ranging snakes from the southeastern United States are grouped in clusters (tip labels in bold, representing current study samples) followed by the number of sequences represented by each excluding recaptures. The topology tree was inferred by Bayesian and maximum-likelihood methods with the maximum-likelihood tree represented. *M. haemocanis [Eperythrozoon canis]* (PQ480193.1) was used as the outgroup (7). Icons at right indicate the host taxa from which *Mycoplasma* spp. was detected.

**Supplementary Figure 3.** *Mycoplasma* phylogenetic tree based on 16s sequences (167 bp). Representative sequences from IGS clusters were selected for 16s analysis (tip labels in bold, representing current study samples) from free-ranging snakes from the southeastern United States. The topology tree was inferred by Bayesian and maximum-likelihood methods with the maximum-likelihood tree represented. *M. haemocanis [Eperythrozoon canis]* (PQ480193.1) was used as the outgroup (7). Icons at right indicate the host taxa from which *Mycoplasma* spp. was detected.

**Supplementary Figure 4.** Changes in pathogen detection after first capture for recaptured snakes sampled in the southeastern United States from 2021 to 2024.

**Supplementary Figure 5.** Predicted lesion severity score in relation to *Ophidiomyces ophidiicola* (*Oo*) DNA concentration (*Oo* DNA copies per ng DNA extract; back-transformed from standardized z-scores for visualization) based on a Poisson generalized linear mixed-effects model in free-ranging snakes collected from 2021 to 2024 from the southeastern United States.


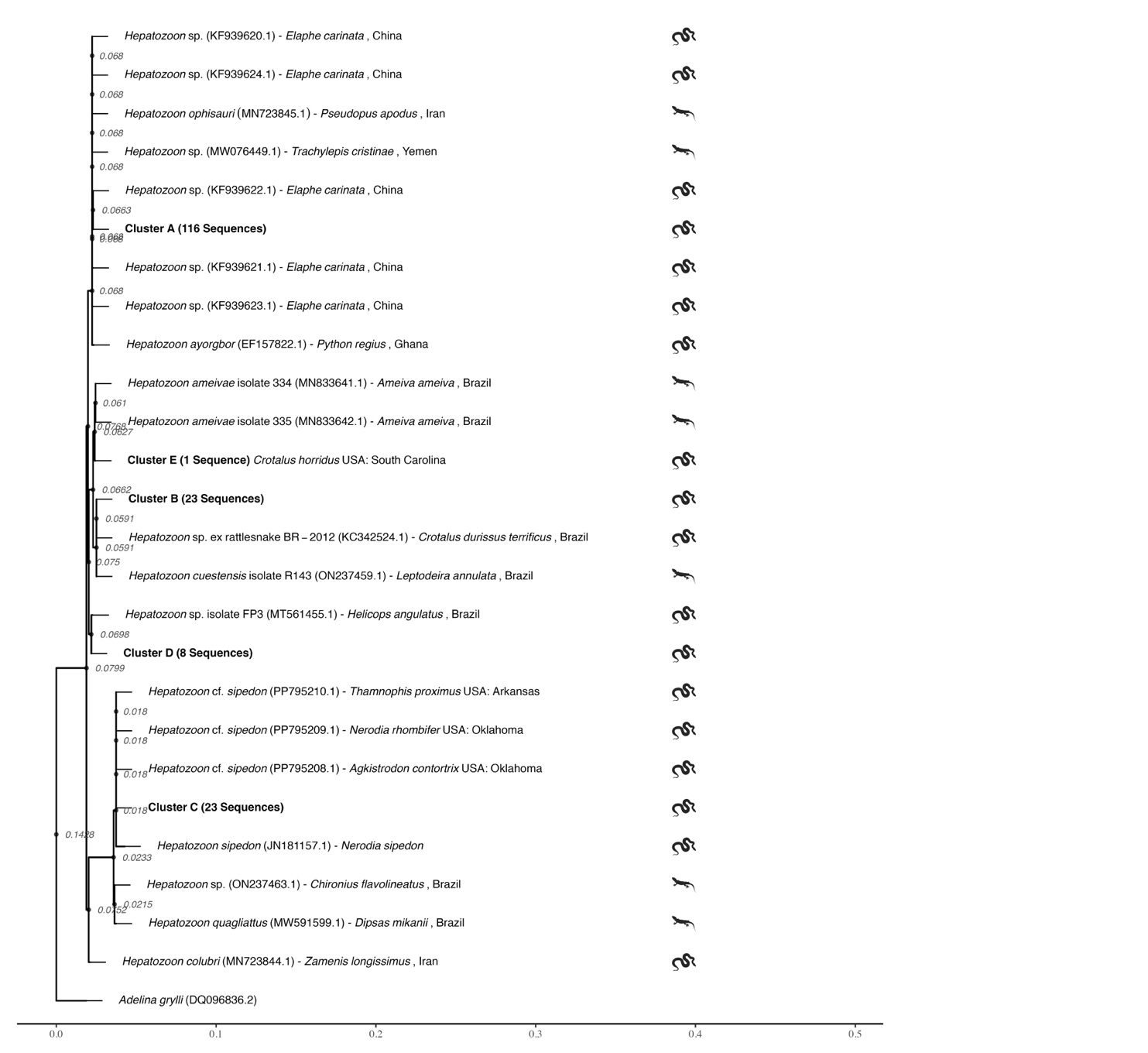


**Supplementary Figure 1.**

*
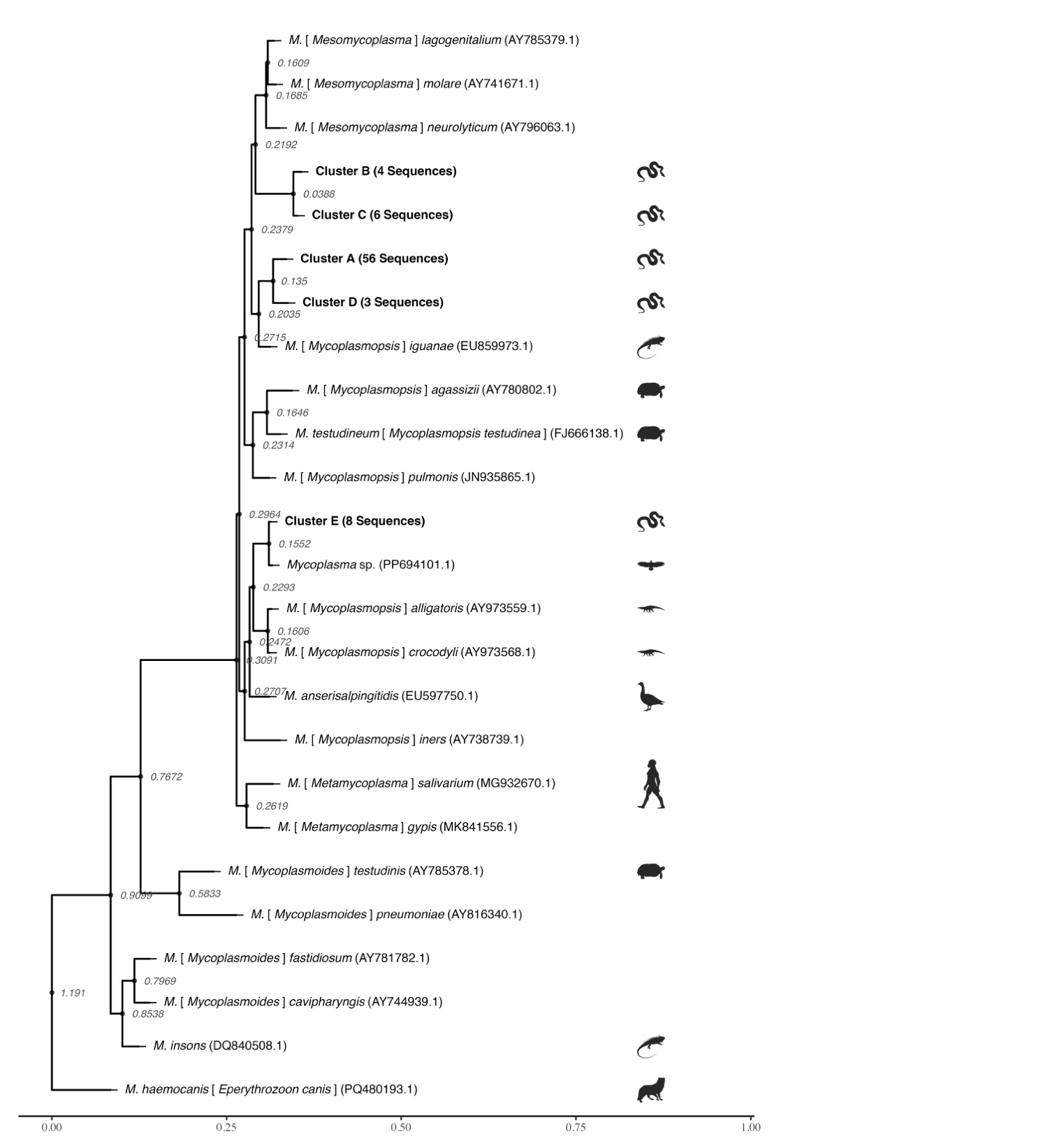
*

**Supplementary Figure 2.**


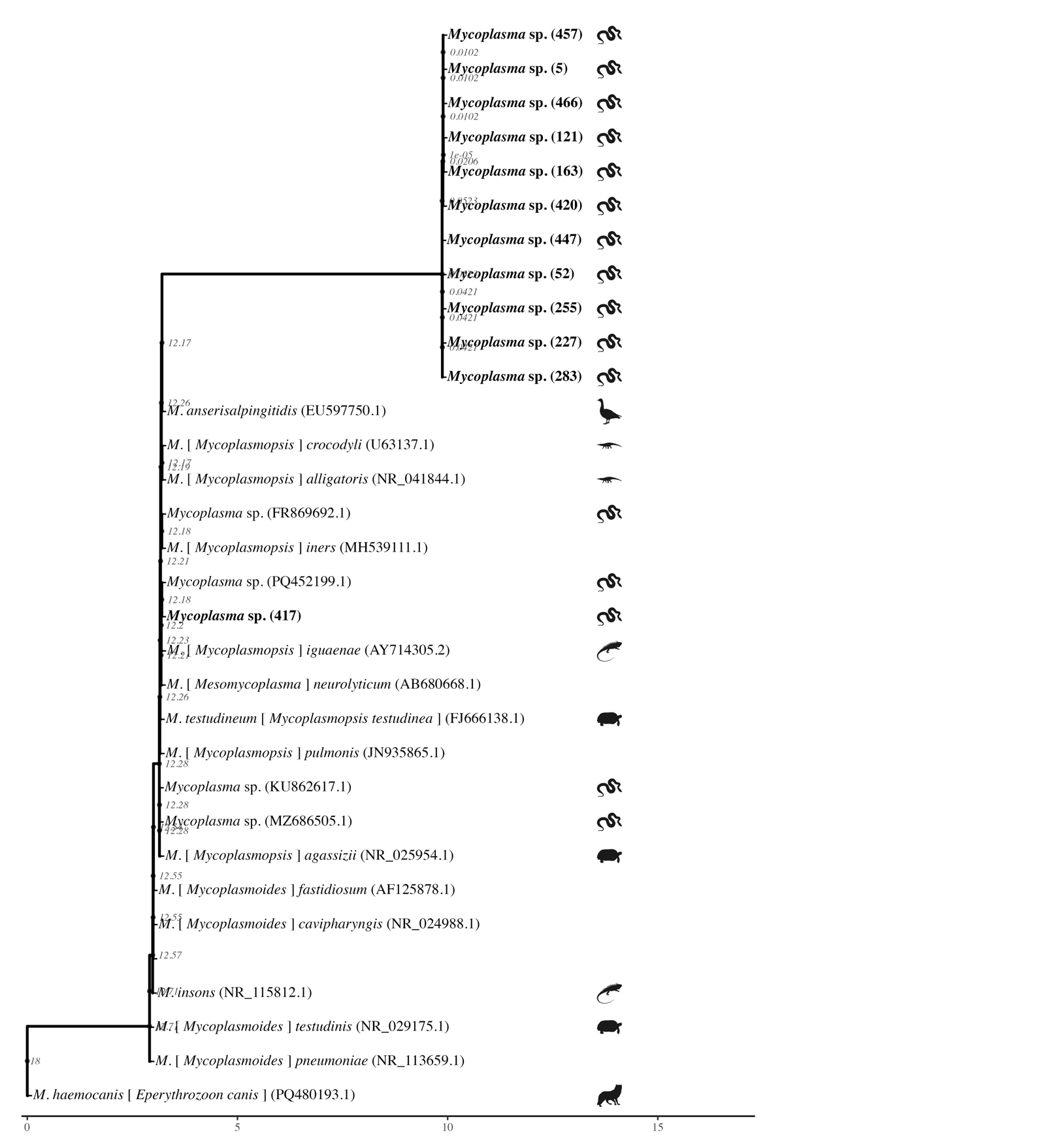


**Supplementary Figure 3.**

**
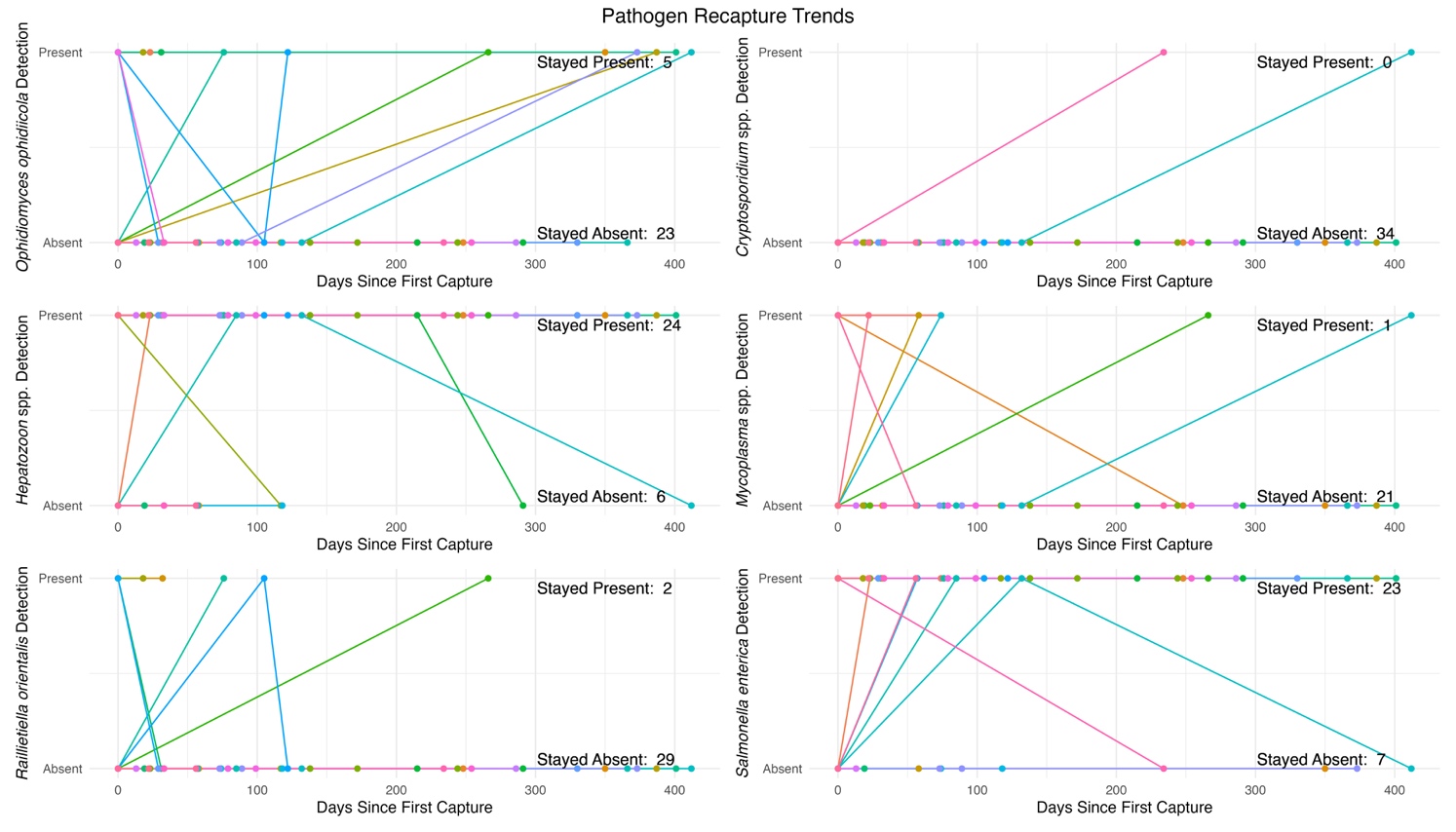
**

**Supplementary Figure 4.**

**
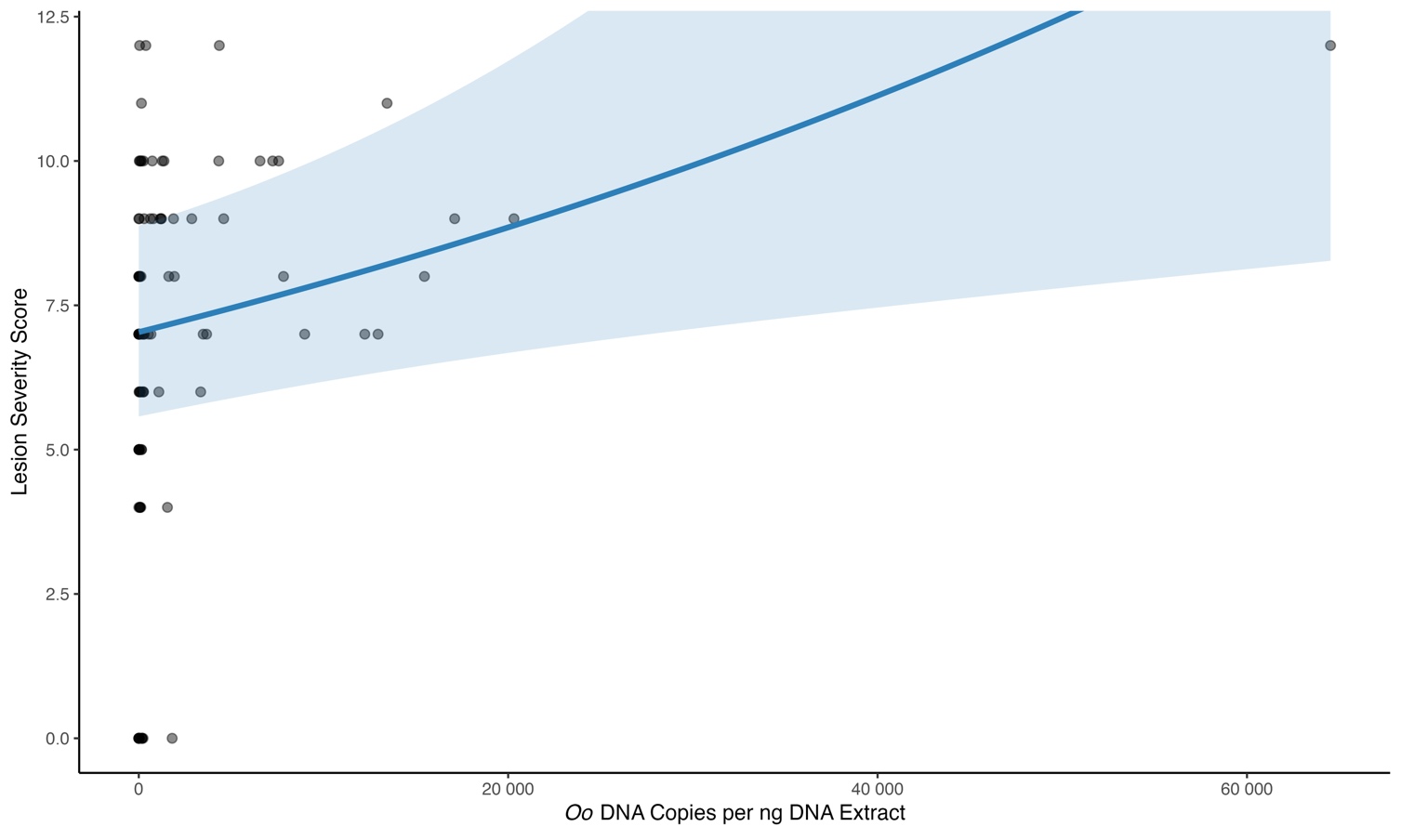
**

**Supplementary Figure 5.**

**Supplementary References**

1. Geneious Prime. 2025.1.1 ed.

2. Edgar RC. MUSCLE v5 enables improved estimates of phylogenetic tree confidence by ensemble bootstrapping. bioRxiv: Cold Spring Harbor Laboratory; 2021.

3. Stamatakis A. RAxML version 8: a tool for phylogenetic analysis and post-analysis of large phylogenies. Bioinformatics. 2014;30(9):1312-3.

4. Huelsenbeck JP, Ronquist F. MRBAYES: Bayesian inference of phylogenetic trees. Bioinformatics. 2001;17(8):754-5.

5. National Center for Biotechnology Information (NCBI) [Internet]. National Library of Medicine (US), National Center for Biotechnology Information. 1988. Available from: <https://www.ncbi.nlm.nih.gov/>.

6. Bates D, Mächler M, Bolker B, Walker S. Fitting linear mixed-effects models using lme4. Journal of Statistical Software,. 2015;67(1):1-48.

7. Gearty W, Jones LA. rphylopic: An R package for fetching, transforming, and visualising PhyloPic silhouettes. Methods in Ecology and Evolution. 2023;14(11):2700-8.
